# Transforming growth factor-β signalling regulates protoscolex formation in the *Echinococcus multilocularis* metacestode

**DOI:** 10.3389/fcimb.2023.1153117

**Published:** 2023-03-22

**Authors:** Marc Kaethner, Kerstin Epping, Peter Bernthaler, Kilian Rudolf, Irena Thomann, Nadine Leitschuh, Monika Bergmann, Markus Spiliotis, Uriel Koziol, Klaus Brehm

**Affiliations:** ^1^ Consultant Laboratory for Echinococcosis, Institute of Hygiene and Microbiology, University of Würzburg, Würzburg, Germany; ^2^ Laboratory of Microbiology and Biotechnology, Department of Food Technology, Fulda University of Applied Sciences, Fulda, Germany; ^3^ Sección Biología Celular, Facultad de Ciencias, Universidad de la República, Montevideo, Uruguay

**Keywords:** echinococcosis, TGFβ, BMP, protoscolex, brood capsule, differentiation, body axes, development

## Abstract

The lethal zoonosis alveolar echinococcosis (AE) is caused by tumor-like, infiltrative growth of the metacestode larval stage of the tapeworm *Echinococcus multilocularis*. We previously showed that the metacestode is composed of posteriorized tissue and that the production of the subsequent larval stage, the protoscolex, depends on re-establishment of anterior identities within the metacestode germinative layer. It is, however, unclear so far how protoscolex differentiation in *Echinococcus* is regulated. We herein characterized the full complement of *E. multilocularis* TGFβ/BMP receptors, which is composed of one type II and three type I receptor serine/threonine kinases. Functional analyzes showed that all *Echinococcus* TGFβ/BMP receptors are enzymatically active and respond to host derived TGFβ/BMP ligands for activating downstream Smad transcription factors. *In situ* hybridization experiments demonstrated that the *Echinococcus* TGFβ/BMP receptors are mainly expressed by nerve and muscle cells within the germinative layer and in developing brood capsules. Interestingly, the production of brood capsules, which later give rise to protoscoleces, was strongly suppressed in the presence of inhibitors directed against TGFβ/BMP receptors, whereas protoscolex differentiation was accelerated in response to host BMP2 and TGFβ. Apart from being responsive to host TGFβ/BMP ligands, protoscolex production also correlated with the expression of a parasite-derived TGFβ-like ligand, EmACT, which is expressed in early brood capsules and which is strongly expressed in anterior domains during protoscolex development. Taken together, these data indicate an important role of TGFβ/BMP signalling in *Echinococcus* anterior pole formation and protoscolex development. Since TGFβ is accumulating around metacestode lesions at later stages of the infection, the host immune response could thus serve as a signal by which the parasite senses the time point at which protoscoleces must be produced. Overall, our data shed new light on molecular mechanisms of host-parasite interaction during AE and are relevant for the development of novel treatment strategies.

## Introduction

Alveolar echinococcosis (AE), a lethal zoonosis prevalent in the Northern Hemisphere, is caused by invasive, tumor-like growth of the metacestode larval stage of the tapeworm *Echinococcus multilocularis* (Thompson, 2017). The infection of the intermediate host (typically rodents, accidentally humans) is initiated by oral uptake of infectious eggs, which contain the parasite’s first larval stage, the oncosphere. Upon hatching within the intestine of the host, the oncosphere penetrates the intestinal wall and gains access to the liver where it undergoes a metamorphosis towards the metacestode (Brehm and Koziol, 2017). Initially, the invading metacestode tissue consists of one or more few fluid-filled, cyst-like structures, with a thin cellular germinal layer (GL) surrounded by an external acellular laminated layer. As we previously showed, parasite proliferation and development strictly depend on a population of pluripotent stem cells (the so-called germinative cells), which are the only mitotically active cells of the parasite and which give rise to all differentiated cells (Koziol et al., 2014). Apart from germinative cells, which account for about 25% of all cell bodies within metacestode tissue, the GL also consists of a syncytial tegument and few differentiated cell types such as storage, muscle, and nerve cells (Brehm and Koziol, 2017). Upon proliferation of metacestode vesicles through budding and invading protrusions, the parasite mass eventually leads to organ failure and host death if not adequately treated (Kern, 2010). Towards the end of the infection in natural intermediate hosts (small rodents), numerous protoscoleces are formed within the GL, which are the head regions of the future adult worm, and which are passed on to the definitive host (foxes, dogs) when it takes the prey. As we previously demonstrated, the ability of tapeworms of the genus *Echinococus* to form cystic structures from the invading oncosphere is due to a modification of the parasite’s body axes (Koziol et al., 2016). According to the current model (Koziol et al., 2016), the parasite’s oncosphere contains a metazoan-typical anterior-posterior body axis formed by posteriorly produced morphogens of the wingless-related (*wnt*) family (such as *wnt-1*), and anteriorly produced *wnt* antagonists such as *sfrp* (secreted frizzled-related protein). During the oncosphere-metacestode transition, the anterior pole is lost, resulting in the posteriorized, invading metacestode, which broadly expresses posterior markers but none of the anterior pole. Upon induction of protoscolex formation, the anterior pole is finally re-established at numerous sites withing the GL, giving rise to protoscoleces with a newly established body axis (Koziol et al., 2016). Whether abandoning and re-establishment of the anterior pole is regulated by parasite-intrinsic signaling pathways, or by host-derived factors, or by both, is currently unknown. Interestingly, protoscolex formation and, thus, re-establishment of the anterior pole, is very rarely seen in human infections (Barth and Casulli, 2021), indicating that host-derived factors might play a role in the modification of the parasite’s body axes during development.

During the infection of the intermediate host, the invading metacestode tissue is subject to several significant environmental changes, which are in part induced by the parasite. Apart from the steadily increasing parasite mass, which may be accompanied by necrosis in distinct regions, the host immune response surrounding the parasite lesions is modified (Mejri et al., 2010). According to current models, the initial establishment of the parasite in rodents is accompanied by a potentially parasitocidal, Th1-dominated immune response with the secretion of elevated levels of interleukin-12 (IL-12), interferon-γ (IFN-g), and tumor necrosis factor-α (Mejri et al., 2010) and at least one parasite-derived factor driving the release of IFN-γ by human T-cells has been identified (Nono et al., 2012). In permissive hosts, the immune response is then gradually shifted towards a permissive Th2-dominated immune response with increased levels of IL-4, IL-5, and IL-10 (Mejri et al., 2010). An important role in inducing an immunosuppressive environment around the parasite at later stages of the disease is exerted by Foxp3+ regulatory T-cells, which secrete high amounts of IL-10 and TGFβ and which are, again, induced by parasite surface structures, metabolites, and excretory/secretory products (Mejri et al., 2010; Nono et al., 2020). In the late chronic phase of AE, high concentrations of host TGFβ are thus accumulating around parasite lesions (Wang et al., 2013).

Cytokines of the TGFβ/bone morphogenetic protein (BMP) superfamily are a phylogenetically ancient group of metazoan cell-cell communication molecules that are involved in many biological processes such as embryonic development, cell proliferation control, and immunoregulation (Massagué, 2012). Based on structural and functional differences, these cytokines are generally separated into the TGFβ/activin branch and the BMP branch, which involve different receptors and downstream signaling molecules. In both cases, serine/threonine surface kinases of the TGFβ/BMP receptor superfamily serve as receptors which, upon cytokine binding and activation, transmit signals to downstream acting Smad transcription factors, the so-called receptor regulated Smads (R-Smads). Upon cytokine binding, TGFβ/BMP receptors usually form tetrameric signaling complexes of type I and type II receptors in which the constitutively active type II receptor phosphorylates, and thereby activates, the type I receptor at a conserved motif (the GS-box) immediately upstream of the kinase domain (Johnson and Newfeld, 2002; Massagué, 2012; Heldin and Moustakas, 2016). In their extracellular regions, both receptor types are structurally similar and contain several highly conserved cysteine residues and a so-called Cys-box motif (CC-X_5-8_-CN), which are important for shaping the ligand binding domain. TGFβ/BMP signaling pathways are best studied in mammals and, in the case of the TGFβ/activin branch, the cytokine first binds to the type II receptor, which then recruits the cognate type I receptor and transmit signals to the AR-branch of R-Smads (e.g. Smad2 or Smad3 in mammals; Johnson and Newfeld, 2002; Massagué, 2012; Heldin and Moustakas, 2016). In the case of BMPs, the cytokine first binds to the type I receptor, which then recruits the type II receptor, resulting in the activation of BR-Smads (e.g. Smad1/5/8 in mammals; Johnson and Newfeld, 2002; Massagué, 2012; Heldin and Moustakas, 2016). Once phosphorylated by the type I receptor at a highly conserved SXS motif at the C-terminal end, the R-Smads then bind to a Co-Smad transcription factor (e.g. Smad4 in mammals) and are transported into the nucleus to activate or repress TGFβ/BMP responsive genes (Johnson and Newfeld, 2002; Massagué, 2012; Heldin and Moustakas, 2016).

We have previously characterized the full complement of *Echinococcus* Smads, consisting of one Co-Smad, EmSmadD, two AR-Smads (EmSmadA, EmSmadC), and two BR-Smads (EmSmadB, EmSmadE), and demonstrated that these factors can be phosphorylated by human TGFβ/BMP type I/type II receptors (Zavala-Góngora et al., 2003; Zavala-Góngora et al., 2008; Epping and Brehm, 2011). Furthermore, we had characterized one *E. multilocularis* member of the BMP receptor family, EmTR1, which, together with the human type II BMP receptor, is activated by human BMP2 and phosphorylates EmSmadB and EmSmadE (Zavala-Góngora et al., 2006). In the present work, we extend our analyzes on *Echinococcus* signaling pathways and characterized the full complement of TGFβ/BMP receptors. We show that these molecules are functionally active kinases, which are expressed in the metacestode and respond to human TGFβ/BMP cytokines. We further demonstrate that TGFβ/BMP signaling is important for the formation of brood capsules (BC) and protoscoleces by the metacestode, which also involves an activin-like cytokine, EmACT, that is produced by the parasite. A model on the induction of BC and protoscolex formation during AE is presented and discussed.

## Materials and methods

### Parasite material and *in vitro* cultivation

All experiments were carried out using the parasite isolates J2012, GH09, and MB17, which derive from Old World Monkey species that had been naturally infected in a breeding enclosure (Tappe et al., 2007). Metacestode tissue was continuously propagated by intraperitoneal passage in Mongolian jirds (*Meriones unguiculatus*) essentially as previously described (Spiliotis and Brehm, 2009). At the time point of these experiments, all isolates were still capable of BC and protoscolex production both *in vitro* and *in vivo*. *In vitro* cultivation under co-culture conditions was carried out as previously described (Spiliotis and Brehm, 2009) using rat Reuber hepatoma cultures as feeder cells. Medium changes were carried out every 3 – 4 days and in cases of inhibitor or cytokine studies, fresh inhibitor or cytokine was added at the indicated concentrations together with medium change. Axenic cultivation was performed using conditioned medium from rat Reuber cells, nitrogen gas phase, and reducing conditions as previously described (Spiliotis et al., 2004; Spiliotis and Brehm, 2009) with medium changes every 3 – 4 days. For inhibitor and cytokine studies, 10 mM stock solutions (in DMSO) of the respective compounds were stored at -80°C and compounds were added to cultures at indicated final concentrations. For negative controls, comparable amounts of DMSO without cytokine/inhibitor were added. Protoscoleces were isolated from *in vivo* cultivated parasite material as previously described (Spiliotis and Brehm, 2009) and activated by low pH/pepsin/taurocholate treatment as described by Ritler et al. (2017).

### Nucleic acid isolation, cloning and sequencing

Total RNA was isolated from *in vitro* cultivated metacestode vesicles using the RNEasy kit (Qiagen, Hilden, Germany) according to the manufacturer’s instructions and cDNA was generated using oligonucleotide CD3-RT essentially as previously described (Hemer and Brehm, 2012). PCR products were cloned employing the PCR cloning kit (Qiagen, Hilden, Germany) or the TOPO TA cloning kit (Thermo Fisher Scientific) and subsequently sequenced by the Sanger method using primers binding to vector sequences adjacent to the cloning sites. For 5’- RACE (rapid amplification of cDNA ends), a previously generated *E. multilocularis* cDNA library was used (Förster et al., 2019), which had been produced using the SMART RACE cDNA amplification kit (Clontech). In all cases, cDNA regions encoding the intracellular regions of *Echinococcus* receptors were first cloned and sequenced using primer sequences designed after genomic information (Tsai et al., 2013), and the verified sequences were then used to design new primers for 5’-RACE, which were employed together with primers of the SMART RACE kit for the specific amplification of 5’ cDNA ends. The sequences of all primers used for cloning are listed in **Table S1**. The corrected full-length sequences of all genes newly characterized in this study were then submitted to the GenBank database and are available under the accession numbers FM178547 (*Emtr2*), HG004205 (*Emtr3*), and ON911572 (*Emtr4*).

### Heterologous expression in HEK293 T cells and cytokine stimulation

Heterologous expression of human and *Echinococcus* TGFβ/BMP receptors together with parasite R-Smad factors in HEK 293 T cells was performed according to previously established methodology (Zavala-Góngora et al., 2003; Zavala-Góngora et al., 2006; Zavala-Góngora et al., 2008; Epping and Brehm, 2011). Briefly, the full-length reading frames of *Emtr2*, *Emtr3*, and *Emtr4* were cloned (without signal peptide encoding regions) into the pSecTag2 vector system (Invitrogen), leading to translational fusions of the *Echinococcus* receptors with the Ig κ chain secretion signal (N-terminal) as well as the myc epitope and a histidine tag (C-terminal). The resulting plasmids were then introduced into HEK 293 T cells as previously described (Zavala-Góngora et al., 2008) in different combinations of type I and type II receptors. All primer sequences used to clone the *Echinococcus* receptors into pSecTag2 are listed in **Table S1**. The generation of constructs for *Echinococcus* R-Smads as well as human type I and type II receptors has been described previously (Zavala-Góngora et al., 2003; Zavala-Góngora et al., 2006; Zavala-Góngora et al., 2008; Epping and Brehm, 2011). In cases of receptor stimulation by exogenously added cytokines, 20 nM recombinant human BMP2 or TGFβ (both by Abcam) were added for 6 h prior to the preparation of cell lysates. Cell lysates were subsequently prepared, separated by SDS-PAGE, and subjected to western blot analysis as described previously (Zavala-Góngora et al., 2003; Zavala-Góngora et al., 2006; Zavala-Góngora et al., 2008; Epping and Brehm, 2011). Antibodies for the detection of phosphorylated R-Smads (purchased from Cell Signaling Technology) were originally directed against human phospho-Smad1/5/8, in the case of EmSmadB and EmSmadE, or against human phospho-Smad2/3 in the case of EmSmadA and EmSmadC and were shown previously to also specifically interact with parasite R-Smads (Zavala-Góngora et al., 2003; Zavala-Góngora et al., 2006; Zavala-Góngora et al., 2008; Epping and Brehm, 2011). As a loading control for comparable amounts of total R-Smad, an antibody directed against the myc-epitope (New England Biolabs) was used. All experiments were performed in triplicates.

### *In situ* hybridization, immunohistochemistry and EdU labelling

Whole-mount *in situ* hybridization (WISH) was performed on *in vitro* cultivated metacestode vesicles with or without brood capsules according to a previously established protocol (Koziol et al., 2014; Koziol et al., 2016). Digoxygenin (DIG)-labeled probes were synthesized by *in vitro* transcription with T7 and SP6 polymerase (New England Biolabs) using the DIG RNA labelling kit (Roche) from cDNA fragments cloned into vector pJET1.2 (Thermo Fisher Scientific). The amplification primers for all probes used in this study are listed in **Table S1**. After hybridization, all fluorescent specimen were processed and analyzed essentially as described recently (Koike et al., 2022). Control experiments using labeled sense probes were always negative. *In vitro* staining of S-phase stem cells was carried out as previously described (Koziol et al., 2014) using 50 µM 5-ethynyl-2’-deoxyuridine (EdU) for a pulse of 5 h after vesicle isolation, followed by fluorescent detection with Alexa Fluor 555 azide as described previously (Koike et al., 2022). Immunohistochemistry against acetylated α-Tubulin (AcTub; antibody from Santa Cruz Biotechnology) and muscular tropomyosin was performed essentially as previously described (Koziol et al., 2014; Koziol et al., 2016). Imaging was performed using a Nikon Eclipse Ti2E confocal microscope.

### RT-qPCR

Total RNA was isolated from vesicles from co-culture and from axenically cultivated vesicles as described above and cDNA was subsequently synthesized as described previously (Koziol et al., 2014) using 100 ng total RNA as input. qPCR was then performed according to a previously established protocol (Pérez et al., 2019; Ancarola et al., 2020) on a StepOne Plus Realtime PCR cycler (Applied Biosystems) using 1 x HOT FIREPol EvaGreen qPCR mix and 300 nM of each primer. Primer sequences for all genes analyzed in this study are listed in **Table S1**. The constitutively expressed gene *elp* (EmuJ_000485800; Ancarola et al., 2020) was used as a control. Cycling conditions were 15 min at 95°C, followed by 40 cycles of 15 sec at 95°C, 20 sec of 58°C, and 20 sec of 72°C. PCR efficiencies were calculated using LinRegPCR (Untergasser et al., 2021), amplification product specificity was assessed by melting curve analysis and gel electrophoresis. Expression levels were calculated by the efficiency correction method using cycle threshold (Ct) values according to Ancarola et al. (2020).

### Bioinformatic procedures and statistical analyzes

Iterative BLASTP searches were carried out against the *E. multilocularis* genome (version 5, January 2016; Tsai et al., 2013) on WormBase ParaSite (Howe et al., 2016) as well as against the SwissProt database as available at GenomeNet (https://www.genome.jp/). For BLASTP searches against *Schistosoma mansoni*, chromosomal assembly version 9 (GCA_000237925.5) was used (Protasio et al., 2012), in the case of *Schmidtea mediterranea*, assembly version ASM260089v1 (Rozanski et al., 2019), all as available under WormBase ParaSite (https://parasite.wormbase.org/index.html). Protein domain structure analysis was carried out using SMART 8.0 (http://smart.embl-heidelberg.de/). Multiple sequence alignments were performed using Clustal Omega (https://www.ebi.ac.uk/Tools/msa/clustalo/) and MEGA11 was used for phylogenetic inference using the maximum likelihood method. Statistical analyzes were performed using GraphPad Prism (version 9) employing one-way t-test or one-way ANOVA (as indicated). For statistical analyzes on positive signals in WISH, 5 randomly chosen fields (380 x 380 µm) per vesicles were counted to obtain a median value and statistical analysis was performed on biological triplicates from different vesicles. For signal counting, 100 randomly chosen nuclei (blue channel) were selected and positive signals for gene specific probes or EdU were calculated on the selected nuclei. For statistical analyzes on BC containing vesicles, average numbers of positive vesicles (10 per well) were determined, and statistics were performed on values from different wells (in biological triplicates).

## Results

### Characterization of *E. multilocularis* TGFβ/BMP receptors

**Figure 1:**
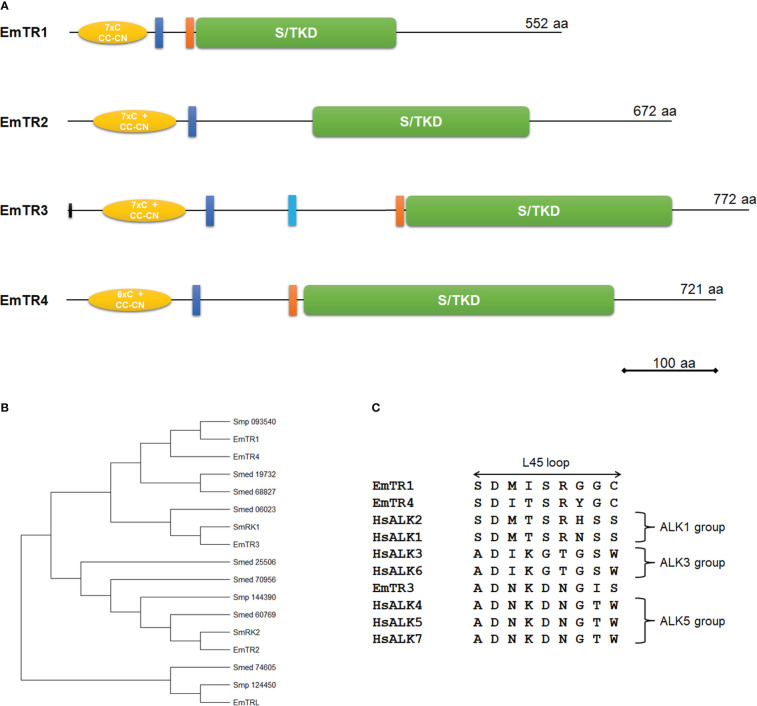
Structure and sequence features of *E. multilocularis* TGFβ/BMP receptors. **(A)** Receptor domain structures. Displayed are structurally important domains for all four characterized TGFβ/BMP receptors. Indicated are the serine/threonine kinase domain (green, S/TKD), the extracellular Cys-rich regions (yellow, with numbers of cysteine residues and CC-CN box), the transmembrane domain (dark blue), and the GS box (orange). The second predicted transmembrane domain for EmTR3 is indicated in light blue. Receptor sizes are indicated to the right. Bar indicates 100 amino acids. **(B)** Phylogenetic tree of different flatworm TGFβ/BMP receptors. Phylogenetic tree has been performed using MEGA software (version 11), applying the Maximum Likelihood method. Indicated are all characterized *Echinococcus* receptors as well as proteins from *S. mansoni* (Smp) and *S. mediterranea* (Smed) according to accession numbers as referenced in **Table S1**. **(C)** Amino acid sequence comparison of L45 loop regions. Displayed are the L45 loop sequences of the *Echinococcus* type I receptors EmTR1, EmTR3, and EmTR4 and human TGFβ/BMP type I receptors (for accession numbers see **Table S1**). Receptor groups are indicated to the right.

To characterize the *E. multilocularis* TGFβ/BMP receptor complement, we first carried out iterative BLASTP analyzes on the available genome information (Tsai et al., 2013). To this end, the amino acid sequences of EmTR1 (Zavala-Góngora et al., 2006), two *S. mansoni* TGFβ receptors (Davies et al., 1998; Beall and Pearce, 2001; Osman et al., 2006), and all known human TGFβ/BMP receptors (Heldin and Moustakas, 2016) were used as queries in BLASTP analyzes against the *E. multilocularis* genome. For all resulting hits with an e-value lower than 10^-4^, predicted amino acid sequences were then extracted and used again for BLASTP analyzes on the *E. multilocularis* genome, to check for potential gene models below the initial threshold, as well as against the SwissProt database, to analyze homologies to mammalian TGFβ/BMP receptors. Furthermore, all predicted sequences were analyzed using SMART (Letunic et al., 2021) to check for the presence of transmembrane and Pfam domains. Only gene models predicted to encode protein kinases were further investigated and subjected to cDNA analysis by PCR and 5’ RACE. Apart from the previously characterized type I receptor EmTR1 (Zavala-Góngora et al., 2006), we thus identified two additional members of the TGFβ/BMP type I receptor family, EmTR3 and EmTR4, as well as one member of the TGFβ type II receptor family, EmTR2 (**Figure 1**).

In the case of EmTR2, the respective gene model (EmuJ_000817800) contained wrong predictions at the 5’ and the 3’ cDNA end. The correct cDNA sequence, as determined by RT-PCR and RACE, encoded a protein of 672 amino acids with predicted transmembrane and protein kinase domains (**Figure 1**; **Figure S1**). As in the case of previously characterized flatworm TGFβ receptors (Davies et al., 1998; Beall and Pearce, 2001; Osman et al., 2006; Zavala-Góngora et al., 2006), no canonical signal peptide was identified. In the extracellular domain, EmTR2 contained a characteristic Cys-box preceded by 7 cysteine residues. A GS-box typical for type I receptors was absent in EmTR2. We thus concluded that EmTR2 encodes a member of the TGFβ/BMP type II receptor family. In the case of EmTR3 we also identified errors in the respective gene model (EmuJ_000758400), which is longer at the 5’ end than predicted. The gene encodes a protein of 772 amino acids with a predicted protein kinase domain and, interestingly, two putative transmembrane domains (**Figure 1**; **Figure S1**). Since EmTR3 is a functionally active receptor (see below), we assume that only one of these predicted regions serves as a true transmembrane domain and, according to sequence comparisons with other receptors (**Figure S1**), this is the more N-terminally located sequence. EmTR3 thus contains a longer juxtamembrane region than other receptors which contains a hydrophobic stretch of yet unknown function. Within the extracellular region, EmTR3 contained a typical Cys-box, preceded by 7 cysteine residues and immediately adjacent to the kinase domain, the protein exhibited a GS box. We thus concluded that EmTR3 is a member of the TGFβ/BMP type I receptor family. Finally, we identified a fourth *E. multilocularis* TGFβ/BMP receptor family member, EmTR4. Again, the respective gene model (EmuJ_000800500) was incorrectly predicted and the coding sequence was longer at the 5’ end. The gene encodes a protein of 721 amino acids with a domain structure very similar to those of EmTR1 and EmTR3. Apart from predicted protein kinase and transmembrane domains, EmTR4 contained structures typical for TGFβ/BMP type I receptors such as an intracellular GS box as well as an extracellular Cys box, preceded by 6 cysteine residues. In total, we thus identified three type I receptors (EmTR1, EmTR3, EmTR4) and one type II receptor (EmTR2) as the full *E. multilocularis* TGFβ/BMP receptor complement. It should be noted that we also identified a fifth gene encoding a protein with homologies to TGFβ/BMP receptors, particularly within the extracellular region. The corresponding gene model (EmuJ_000906600) predicts a protein with a characteristic Cys box and 7 additional cysteine residues in the extracellular domain, followed by a transmembrane domain. SMART analyzes did not, however, identify a protein kinase domain and when we compared the deduced amino acid sequence with those of the other *Echinococcus* receptors, we found that it only contained one of six invariant and highly conserved kinase domain residues (Hammarén et al., 2015) that are essential for the catalytic process (**Figure S1**). We thus concluded that this protein is most likely not an active protein kinase and termed the protein EmTRL (for *E. multilocularis* TGFβ receptor-like protein).

To analyze phylogenetic relationships between the identified *Echinococcus* receptors and previously characterized TGFβ receptor family members from the related flatworm *S. mansoni* (Davies et al., 1998; Beall and Pearce, 2001; Osman et al., 2006), we then carried out iterative BLASTP searches against the *S. mansoni* genome as available in WormBase ParaSite. We also included in these studies the genome of the related planarian *Schmidtea mediterranea*, in which TGFβ signalling is involved in size-dependent fission regulation (Arnold et al., 2019). For *S. mansoni* we identified five receptors, of which two (Smp_049760, formerly described as SmRK1 (Beall and Pearce, 2001); Smp_093540) contained a GS-box that is characteristic of type I receptors, whereas three others (Smp_334370, formerly described as SmRK2 (Forrester et al., 2004); Smp_144390, Smp_124450) lacked GS-boxes and, therefore, represented type II receptors. In *S. mediterranea* we identified a total of seven potential receptors, represented by gene models SMESG000006023, SMESG000019732, and SMESG000068827 for potential type I receptors with GS-box, and SMESG000060769, SMESG000070956, SMESG000025506, as well as SMESG000074605 for type II receptors. As shown in the phylogenetic tree in **Figure 1B**, within the type I receptor branch EmTR3 grouped together with SmRK1, which together with SmRK2 transmits TGFβ signals in *S. mansoni* (Osman et al., 2006). Within the type II receptor branch, EmTR2 is closely related to SmRK2, indicating that the *Echinococcus* equivalent to the schistosome receptor pair SmRK1/SmRK2 is formed by EmTR3 and EmTR2. The type I receptor EmTR4 shows highest similarity to the previously characterized EmTR1, which transmits BMP signals in *Echinococcus* (Zavala-Góngora et al., 2006), and both are grouped together with schistosome Smp_093540, which has not yet been characterized biochemically. These data indicate that at least on the level of overall sequence homologies EmTR1 and EmTR4 belong to the BMP type I receptor branch whereas EmTR3 probably transduces TGFβ/activin signals. Interestingly, within the phylogenetic tree EmTRL forms an outgroup together with Smp_124450 and the planarian receptor Smed_74605 (**Figure 1**), which both contain protein kinase domains as predicted by SMART (data not shown). This indicates that EmTRL shares a common protein kinase containing ancestor with Smp_124450 but has subsequently lost amino acid residues that are crucial for protein kinase activity.

The interaction between members of the TGFβ/BMP receptor families and downstream R-Smads is governed by two elements, called the L45 loop in the case of the receptors, and the L3 loop in the C-terminal region of the R-Smads (Persson et al., 1998). We had previously characterized several *Echinococcus* R-Smads of which two (EmSmadA, EmSmadC) belong to the AR-Smad group, which usually transmit TGFβ/activin signals, and two others (EmSmadB, EmSmadE) to the BR-Smads, that transmit BMP signals (Epping and Brehm, 2011). To assess which of the identified type I receptors potentially interacts with *Echinococcus* Smads we thus closer analyzed the respective L45 loop sequences (**Figure 1**) and found close similarities between the L45 loops of EmTR1 and EmTR4 to the ALK1 receptor group, which usually interacts with BR-Smads (Massagué, 1998). EmTR3, on the other hand, showed similarity to the ALK5 receptor group, which transmits signals to AR-Smads (Massague et al., 1998). In previous analyzes we had already shown that EmTR1 can phosphorylate EmSmadB and EmSmadE (Zavala-Góngora et al., 2006; Epping and Brehm, 2011), but not EmSmadA or EmSmadC (Zavala-Góngora et al., 2006; Zavala-Góngora et al., 2008). Consequently, we would expect that EmTR4 also interacts with EmSmadB and EmSmadE, whereas EmTR3 should transmit signals *via* EmSmadA and/or EmSmadC.

Taken together, our analyzes so far showed that the *E. multilocularis* complement of TGFβ/BMP receptors consists of three type I receptors, of which two (EmTR1, EmTR4) belong to the BMP- and one (EmTR3) to the TGFβ/Activin subfamilies, and only one type II receptor (EmTR2) with a canonical protein kinase domain. EmTRL, on the other hand, probably derived from a functional type II receptor, but during evolution has lost residues that are important for kinase activity.

### Functional activities of *E. multilocularis* TGFβ/BMP receptors

Next, we were interested in whether the identified *E. multilocularis* TGFβ/BMP receptors are functionally active and whether EmTR2 might act as a cognate type II receptor for EmTR1, EmTR3, and EmTR4. To this end, we used heterologous expression of human and *Echinococcus* TGFβ/BMP receptors in HEK293 T cells together with all *Echinococcus* R-Smads. The assay relies on the co-expression of type I and type II receptors and, as a readout for enzymatic activity, phosphorylation of co-expressed R-Smads at two C-terminal serine residues within a conserved SXS motif is measured employing phospho-specific antibodies.

**Figure 2:**
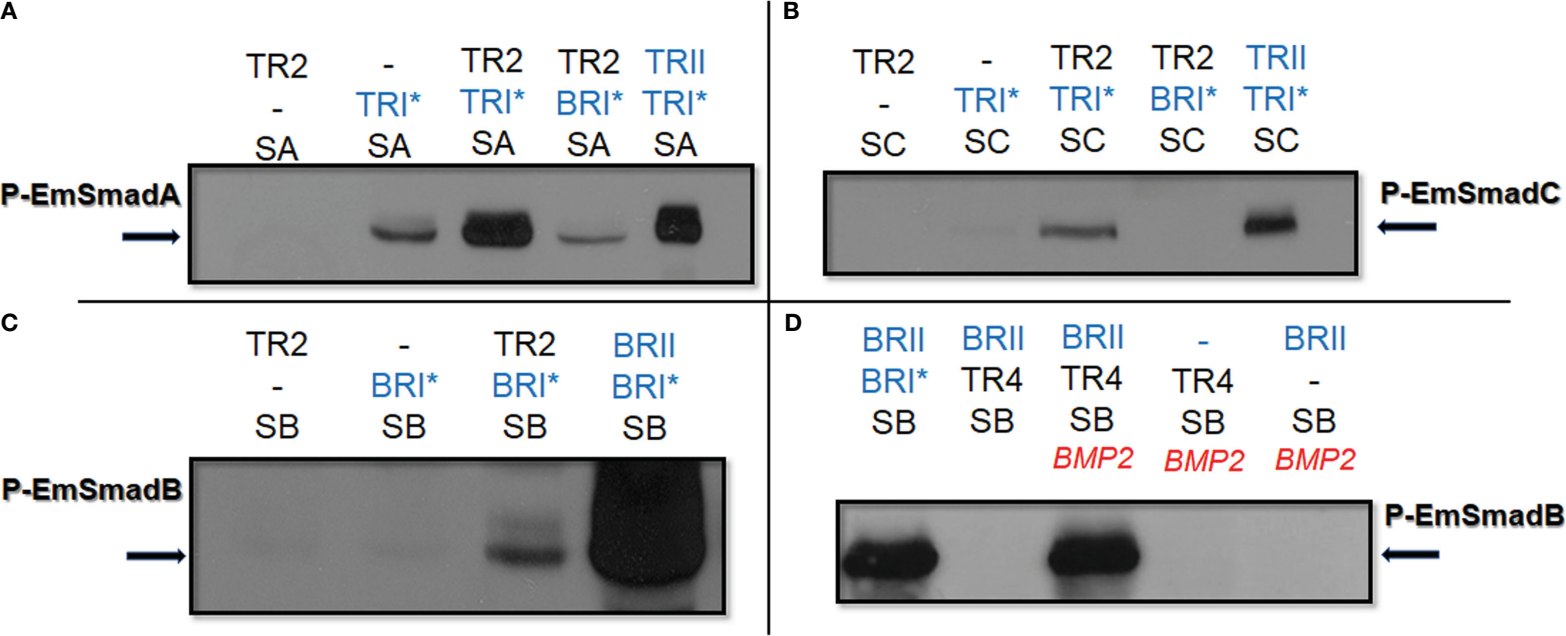
Functional interaction of human and *Echinococcus* TGFβ/BMP receptors. *Echinococcus* and human TGFβ/BMP receptors were co-expressed with *Echinococcus* R-Smads in HEK293 T cells either with or without stimulation by exogneous human cytokines. Cell lysates were subjected to PAGE and Western blot analysis using phospho-specific antibodies. Displayed are the results for EmSmadA **(A)**, EmSmadC **(B)**, and EmSmadB **(C)** upon co-expression with EmTR2 and different human TGFβ/BMP receptors (as indicated). In **(D)**, results for EmSmadB upon co-expression with EmTR4 and the human type II BMP receptor (BRII) are shown. Human type II receptors for BMP (BRII) as well as constitutively activated type I receptors for BMP (BRI*) and TGFβ (TRI*) are shown in blue. Abbreviations for Echinococus receptors (in black) indicate EmTR2 (TR2) and EmTR4 (TR4), abbreviations for R-Smads are SA (EmSmadA), SB (EmSmadB), and SC (EmSmadC). The addition of exogenous human BMP2 is indicated in red. Arrows to the left and right indicate the phosphorylated R-Smad forms. In all cases comparative amounts of total R-Smads, as measured using an anti-Myc-tag antibody, were applied to the gel. Results show representative examples of experiments performed in triplicates. -, not added.

**Figure 3:**
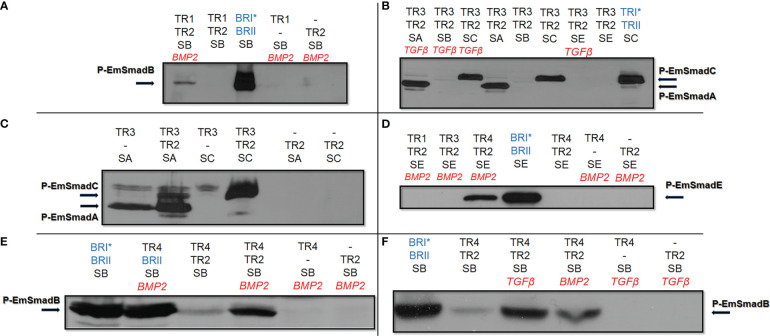
Functional activities of Echinococcus TGFβ/BMP receptors. *Echinococcus* type I and type II TGFβ/BMP receptors were co-expressed with *Echinococcus* R-Smads in HEK 293 T cells either with or without stimulation by exogneous human cytokines. Cell lysates were subjected to PAGE and Western blot analysis using phospho-specific antibodies. Displayed are the results for EmTR1 and EmTR2 on EmSmadB **(A)**, EmTR3 and EmTR2 on EmSmadA and EmSmadC **(B)**, EmTR3 and EmTR2 on EmSmadA and EmSmadC **(C)**, all *Echinococcus* type I receptors with EmTR2 on EmSmadE **(D)**, as well as EmTR4 and EmTR2 on EmSmadB upon stimulation with exogenous BMP2 **(E)** or TGFβ **(F)**. Human type II receptors for BMP (BRII) and TGFβ (TRII) as well as constitutively activated forms of human type I receptors for BMP (BRI*) or TGFβ (TRI*) were included as controls (shown in blue). Abbreviations for *Echinococus* receptors (in black) indicate EmTR1 (TR1), EmTR2 (TR2), EmTR3 (TR3), and EmTR4 (TR4), abbreviations for R-Smads are SA (EmSmadA), SB (EmSmadB), and SC (EmSmadC), and SE (EmSmadE). The addition of exogenous human BMP2 or TGFβ is indicated in red. Arrows to the left and right indicate the phosphorylated R-Smad forms. In all cases comparative amounts of total R-Smads, as measured using an anti-Myc-tag antibody, were applied to the gel. Additional bands in **(C, E)** are background deriving from endogenously expressed Smads of HEK 293 T cells. Results show representative examples of experiments performed in triplicates. -, not added.

In our previous work we had already shown that EmTR1 can form an enzymatically active receptor pair with the human BMP type II receptor to phosphorylate the BR-Smads EmSmadB and EmSmadE (Zavala-Góngora et al., 2006; Epping and Brehm, 2011). The AR-Smads EmSmadA and EmSmadC, on the other hand, were not phosphorylated by EmTR1 (Zavala-Góngora et al., 2006, Zavala-Góngora et al., 2008). In the present work, we first investigated whether EmTR2 can serve as cognate receptor for human type I receptors and employed the activated forms of these receptors, BRI* (Q223 mutation) and TRI* (T204D mutation), which require a cognate type II receptor for full activity, but act independently of exogenous TGFβ/BMP ligand (Hassel et al., 2003). As shown in **Figure 2A**, *Echinococcus* EmSmadA was already phosphorylated to a certain extent by human TRI* in the absence of a type II receptor, but EmSmadA phosphorylation was significantly stimulated in the presence of EmTR2. Furthermore, EmSmadC was exclusively phosphorylated by human TRI* upon co-expression with EmTR2 (**Figure 2B**). Likewise, when tested in combination with human BRI* we only observed phosphorylation of EmSmadB when the human receptor was co-expressed with EmTR2 (**Figure 2C**). Taken together, these analyzes indicated that EmTR2 is a functionally active type II receptor and can act in combination with human TGFβ/BMP type I receptors to phosphorylate R-Smads. In the case of EmTR3, we already observed phosphorylation of *Echinococcus* EmSmadA when expressed without any type II receptor, clearly indicating that it is an active kinase (**Figure 3C**). Finally, EmTR4 was able to phosphorylate EmSmadB upon co-expression with human BMPRII, but only when stimulated by exogenous addition of human BMP2. These data indicated that EmTR4 is not only an active kinase, but, like EmTR1, also directly interacts with human cytokine.

Next, we investigated whether EmTR2 can serve as a cognate type II receptor for the *Echinococcus* type I receptors. As shown in **Figure 3A**, co-expression of EmTR1 with EmTR2 resulted in the phosphorylation of EmSmadB, which required stimulation with exogenous BMP2. The second *Echinococcus* BR-Smad, EmSmadE, however, was not phosphorylated by EmTR1/EmTR2 under these conditions (**Figure 3D**). We then co-expressed EmTR3 with EmTR2 in the presence of all *Echinococcus* R-Smads with or without stimulation by human TGFβ. As shown in **Figure 3B**, this led to phosphorylation of the AR-Smads EmSmadA and EmSmadC, but the *Echinococcus* BR-Smads EmSmadB and EmSmadE were not recognized by EmTR3. Interestingly, the functional interaction between EmTR3 and EmTR2 operated independently of exogenous TGFβ (**Figure 3B**). To investigate whether EmTR3 at least requires the presence of EmTR2 for Smad activation, we then expressed the type I receptor either alone or together with the type II receptor in the presence of R-Smads. As shown in **Figure 3C**, the phosphorylation of EmSmadA with EmTR3 alone was clearly detectable but was further stimulated in the presence of EmTR2. In the case of EmSmadC, however, both receptors were necessary for R-Smad phosphorylation (**Figure 3C**). These results indicated that EmTR2 also serves as a type II receptor for stimulating the activity of EmTR3 towards the *Echinococcus* AR-Smads. Finally, we conducted similar experiments on EmTR4. As shown in **Figure 3D**, EmTR4 phosphorylated EmSmadE, but required the presence of EmTR2 and exogenous stimulation by human BMP2. Likewise, EmTR4 also phosphorylated EmSmadB in the presence of EmTR2, which was stimulated by exogenous BMP2 (**Figure 3E**). Interestingly, EmTR4 also activated EmSmadB when stimulated by human TGFβ (**Figure 3F**). These data indicated that EmTR4 forms a functional receptor complex with EmTR2, and that this combination is responsive to both human BMP2 and TGFβ.

Taken together, our combined type I/type II receptor experiments indicated that (i) all *Echinococcus* TGFβ/BMP receptors are functionally active, (ii) EmTR2 serves as a cognate type II receptor for all *Echinococus* type I receptors, and (iii) *Echinococcus* TGFβ/BMP receptors are responsive to human BMP2 and/or TGFβ.

### Expression of *Echinococcus* TGFβ/BMP receptors in parasite larvae

We next investigated expression patterns of all *Echinococcus* TGFβ/BMP receptors in the metacestode. Previous transcriptomic analyzes that had been carried out during the *E. multilocularis* genome sequencing project (Tsai et al., 2013) indicated that all receptors are well expressed in all larval stages with *Emtr4* having the highest transcript levels, followed by *Emtr1*, *Emtr2*, and *Emtr3* (**Figure S2**). Since *Emtrl* encoded a protein without canonical protein kinase domain and was low abundantly expressed in parasite larvae (**Figure S2**), we only concentrated on genes encoding functionally active kinases. To identify individual metacestode cells which express the receptor genes, we carried out whole mount *in situ* hybridization (WISH). Furthermore, to identify gene expression in parasite stem cells, WISH was combined with staining for EdU incorporating cells (5 h pulse), which specifically labels proliferating stem cells in S-phase (Koziol et al., 2014).

**Figure 4:**
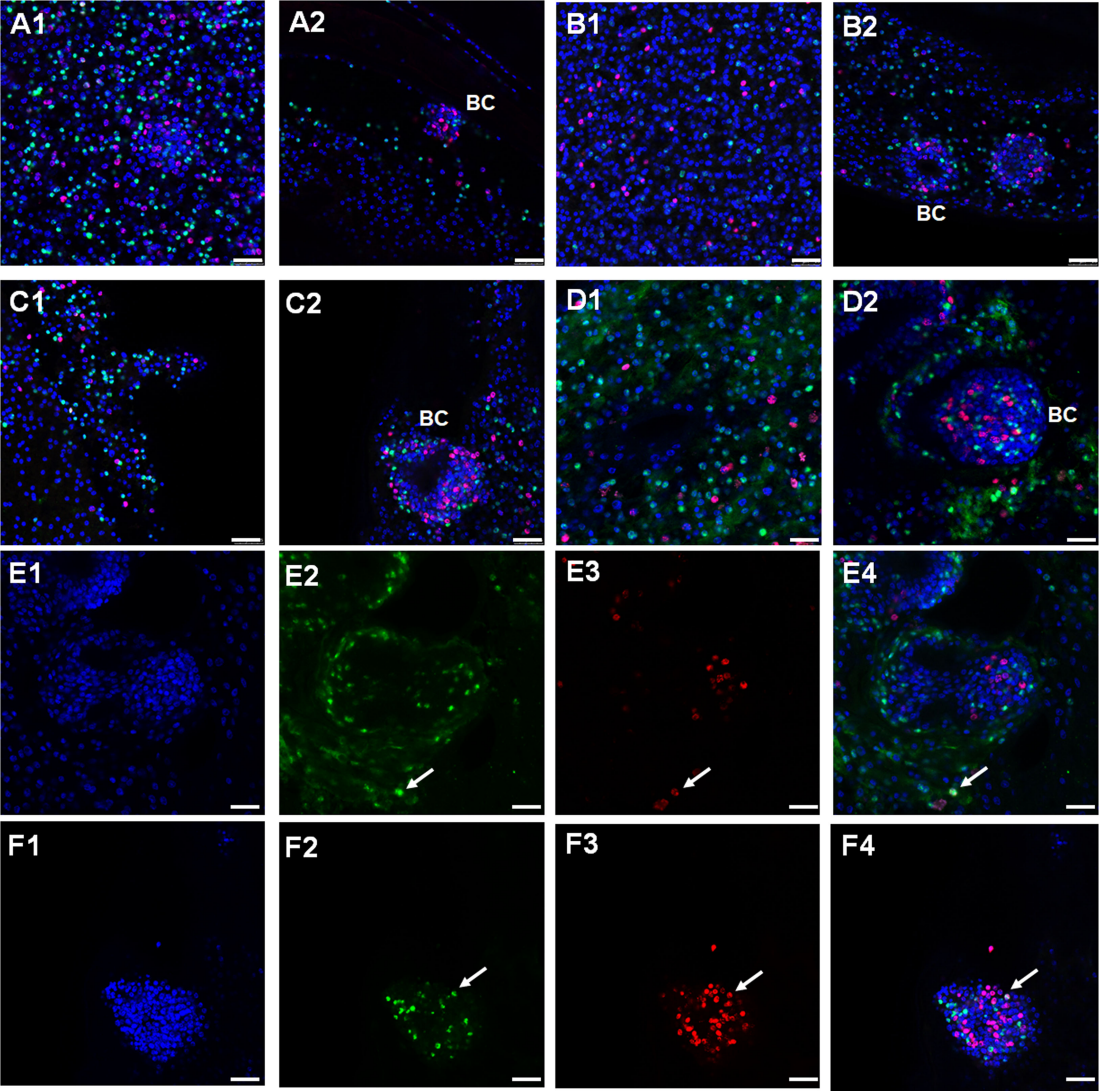
Expression of *Echinococcus* TGFβ/BMP receptor encoding genes in germinative layer and BC. Representative WISH images are shown for *Emtr1* **(A1, A2)**, *Emtr2* **(B1, B2)**, *Emtr3* **(C1, C2)**, and *Emtr4* **(D1, D2)** in the germinative layer **(A1, B1, C1, D1)** and in developing brood capsules **(A2, B2, C2, D2)**. A to D shows merges of all three channels. Brood capsules are indicated by ‚BC’. Examples of cells positive for EdU and for *Emtr4* within the germinative layer **(E1 to E4)** or brood capsules **(F1 to F4)** are marked by a white arrow. These images show separate channels for DAPI (nuclei, blue; **E1, F1**), receptor gene probe (green; **E2, F2**), EdU (S-phase, red; **E3, F3**), and a merge of all three channels **(E4, F4)**. Bar represents 25 µm.

As shown in **Figure 4**, we obtained signals for all receptors, which were distributed over the parasite’s GL without specific pattern. For all receptor encoding genes, we also identified signals in early BC, which later give rise to protoscoleces (**Figure 4**). We then counted the number of cells positive for WISH and for EdU signals and concentrated on the GL since cell counting in BC is difficult due to high cell densities. As reflected in the overall transcriptomic data (**Figure S2**), the highest density of WISH+ cells was found for *Emtr4* (22 ± 3% WISH+ of all cells, n = 3939), followed by *Emtr1* (19 ± 3%, n = 1484) and *Emtr3* (10 ± 2%, n = 2947). The lowest number of WISH+ cells was found for *emtr2* (5 ± 1%, n = 2407), which was unexpected since EmTR2 serves as a cognate type II receptor for EmTR1, EmTR3, and EmTR4 (**Figure 4**). Hence, within the GL there is a considerable portion of cells which only express type I receptors but obviously no cognate type II receptor.

In all vesicles analyzed we counted approximately 5% (of all cells) to be EdU+ cells. Interestingly, in all cases the vast majority of WISH+ signals did not co-localize with EdU, indicating that the *E. multilocularis* TGFβ/BMP receptors are almost exclusively expressed in post-mitotic cells. However, as shown in **Figure 4E** and **Figure 4F**, we also detected few cases of WISH+/EdU+ cells within both the germinative layer and brood capsules. Within the germinative layer, these amounted to approximately 0.4% (of all EdU+ cells) in the case of *Emtr1*, *Emtr3*, and *Emtr4* and to 1.6% in the case of *Emtr2*. Hence, whereas *Emtr2* showed the lowest overall cell numbers of WISH+ signals, it also showed the highest rate of expression in stem cells.

**Figure 5:**
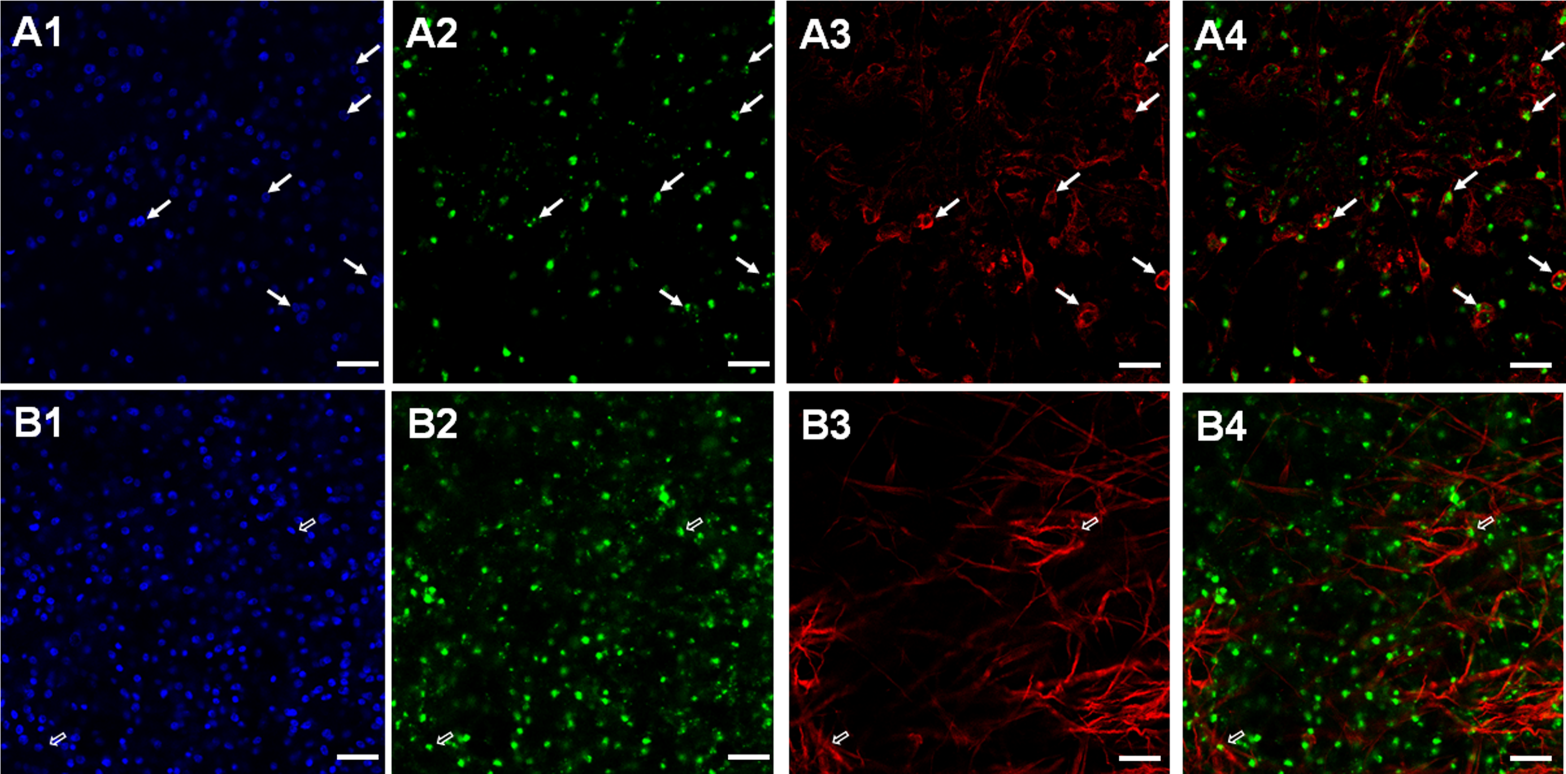
Expression of *Echinococcus Emtr2* in nerve and muscle cells. Representative WISH images are shown for *Emtr2* expression in nerve **(A)** and muscle **(B)** cells. Channels indicate DAPI (nuclei, blue; **(A1, B1)**), *Emtr2*-specific probe (green; **(A2, B2)**), anti-AcTub (nerve cells; **(A3)**), and anti-HMW tropomyosin (muscle fibers; **(B3)**). Merges of all three channels are shown in **(A4, B4)**. Cells double positive for *Emtr2* and AcTub are indicated by white closed arrows, cells positive for *Emtr2* and HMW tropomyosin by open white arrows. Bar indicates 25 µm.

To further elucidate which post-mitotic cell types expressed these receptors, we carried out WISH combined with staining for acetylated α-tubulin (AcTub), which specifically stains nerve cells within the germinal layer (Koziol et al., 2013). In these experiments we concentrated on *Emtr2* since we assumed that functional, cytokine-responsive receptor complexes are exclusively formed between the *Echinococcus* type I receptors and EmTR2 (see above). As shown in **Figure 5A**, we indeed identified many AcTub+ cells which co-stained with *Emtr2*. Due to more diffuse signals in AcTub staining, an exact quantification of co-staining is hard to achieve but we found around 80 – 90% of AcTub+ cells with *Emtr2* signals (**Figure 5**). Since we also found a considerable number of *Emtr2*+ cells, which were not associated with AcTub (**Figure 5A**), we then also analyzed whether the type II receptor is associated with muscle cells, another dominant cell type of the germinative layer (Koziol et al., 2013; Koziol et al., 2016). To this end, we carried out WISH against *Emtr2* combined with staining against muscle cell-specific high molecular weight (HMW) tropomyosin (Koziol et al., 2016) and, as depicted in **Figure 5B**, we found numerous muscle cells which were positive for *Emtr2* although, again, exact quantification was impossible since myofibers are usually very difficult to allocate to their respective muscle cell bodies.

In summary, our WISH experiments indicated that the *E. multilocularis* TGFβ/BMP receptors are predominantly expressed by post-mitotic cells, including nerve and muscle cells of the GL. In very few cases we also found S-phase stem cells expressing TGFβ/BMP receptors, particularly for *Emtr2*.

### Inhibition of *E. multilocularis* TGFβ/BMP receptors prevents protoscolex production

In previous work we showed that small molecule compounds designed to inhibit human kinases also effectively inactivate related cestode enzymes, albeit at higher IC_50_ values (Gelmedin et al., 2008; Hemer and Brehm, 2012; Hemer et al., 2014; Schubert et al., 2014; Förster et al., 2019; Stoll et al., 2021). We were therefore interested whether this approach can be used to study the role of TGFβ/BMP receptors in *Echinococcus* development. As one candidate we chose compound SB431542, which specifically targets mammalian type I receptors of the TGFβ/activin branch (Ogunjimi et al., 2012). Using the HEK293 T cell expression system, we first tested whether SB431542 inhibits *Echinococcus* type I receptors. As shown in **Figure S3**, at 10 μM concentration, SB431542 completely inhibited the activity of EmTR3 and partially that of EmTR4. EmTR1, on the other hand, was not affected by SB431542 at 10 µM (data not shown). This is in line with previous reports showing that the specificity of SB431542 for the TGFβ branch of receptors over BMP type receptors is due to the kinase gatekeeper residue, which is serine in the case of all human TGFβ type I receptors and the larger residue threonine in human BMP receptors (Ogunjimi et al., 2012). Although EmTR4 by sequence homology belongs to the BMP receptor branch, it contains serine as gatekeeper (**Figure S3**), thus explaining why it is inhibited by SB431542. EmTR1, on the other hand, harbors threonine as gatekeeper, which prevents access of the inhibitor to the ATP binding pocket. In addition to SB431542, we also tested the related compound A8301, which also inhibited EmTR3 and partially EmTR4 at a concentration of 10 µM (**Figure S3**). It should be noted that previous studies on TGFβ receptors of schistosomes utilized the compound TRIKI (also known as LY364947), which caused effects on parasite fertility (Buro et al., 2013). In our system, however, TRIKI did not show inhibitory effects against any *Echinococcus* type I receptor at 10 µM (data not shown).

**Figure 6:**
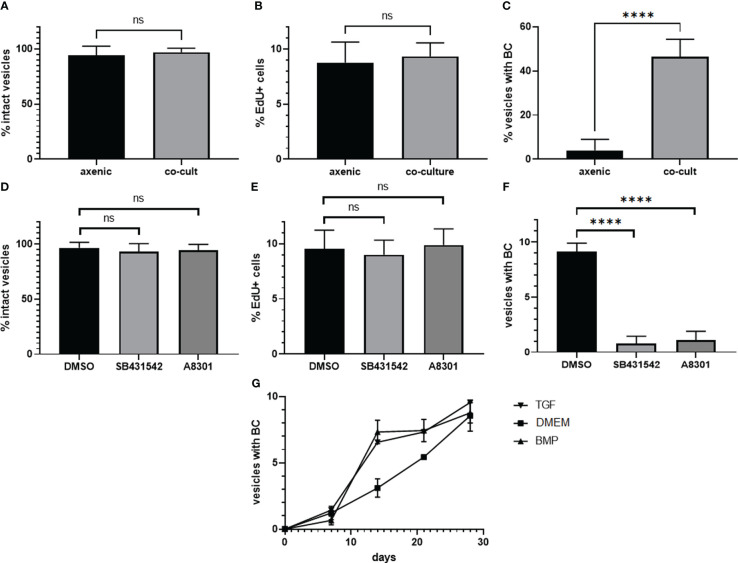
Influence of culture conditions and TGFβ receptor inhibitors on BC formation. Upper panel: Influence of axenic cultivation (axenic) and co-culture with host feeder cells (co-cult) on vesicle survival **(A)**), stem cell proliferation **(B)**, and BC formation **(C)** in metacestode vesicles of isolate MB17. Vesicles were grown *in vitro* under culture conditions as indicated for 3 weeks. Vesicle integrity **(A)** was measured light microscopically, stem cell proliferation **(B)** by EdU incorporation (in % EdU+ cells of all cells), and BC formation by light microscopy (visible BC). DMSO indicates control. Statistical analysis: unpaired t-test in GraphPad Prism. ns, not significant, ****p < 0.0001. Middle panel: Influence of TGFβ receptor inhibitors on metacestode vesicle survival **(D)**, stem cell proliferation **(E)**, and BC formation **(F)**. Metacestode vesicles of isolate MB17 were grown *in vitro* in co-culture in the presence of 10 µM of SB431542 and A8301 (as indicated) with medium changes every 3 - 4 days. Vesicle integrity, stem cell proliferation, and BC formation was assessed as in A – **(C)** DMSO indicates control. Statistical analysis: one-way ANOVA in GraphPad Prism. ns = not significant, ****p < 0.0001. **(G)** BC formation in vesicles in the presence of TGFβ/BMP cytokines. Vesicles of isolate MB17 were *in vitro* cultivated in the co-culture system for 28 days in the presence of 1 nM of recombinant human TGFβ (TGF) or BMP2 (BMP) with medium changes every 3 – 4 days. BC formation was assessed light microscopically. DMEM medium was used as a control. Shown are median with standard deviation. Unpaired t-test as assessed by GraphPad prism showed **** (p < 0.0001) at day 14 between TGFβ and the control as well as between BMP2 and the control.

We next tested whether SB431542 and A8301 affect growth and survival of *in vitro* cultivated metacestode vesicles. For metacestode *in vitro* cultivation we routinely either use a co-culture system in which metacestode tissue is continuously cultivated together with host hepatoma cells (Spiliotis and Brehm, 2009). Alternatively, we employ an axenic cultivation system in which metacestode vesicles are kept in the absence of host cells but under nitrogen atmosphere (Spiliotis et al., 2004; Spiliotis and Brehm, 2009). The axenic cultivation system is our regular approach to test inhibitor effects since it prevents the measurement of indirect effects on co-cultivated host cells (Gelmedin et al., 2008; Hemer and Brehm, 2012; Hemer et al., 2014; Schubert et al., 2014; Förster et al., 2019; Stoll et al., 2021). Since TGFβ signalling is involved in differentiation processes in planarians (Arnold et al., 2019) we were also interested in BC and protoscolex formation by metacestode vesicles. To see whether the culture conditions alone would affect *Echinococcus* protoscolex production, we first measured BC formation under the respective culture conditions for isolate MB17. As shown in **Figure 6**, although axenic culture conditions did not significantly affect vesicle survival and stem cell proliferation when compared to co-cultivation, BC almost exclusively developed in the co-cultivation system. In the following experiments, we therefore concentrated on the co-culture system.

Inhibitor effects on vesicle BC formation are difficult to investigate since depending on the duration of *in vivo* passages in laboratory animals, vesicles of different isolates either start very early with brood capsule formation (when the vesicles are around 3 mm in diameter) or later when vesicles have reached a size of 5 mm or larger. We therefore first screened different *E. multilocularis* isolates available in our laboratory for the capacity to produce brood capsules and protoscoleces *in vitro* and found isolate MB17 particularly suitable since it regularly formed microscopically visible BC within 3 weeks in co-culture when vesicles had reached a size of 5 mm in diameter. We then isolated MB17 vesicles, cultivated them in the presence or absence of inhibitors for 4 weeks, and inspected structural integrity of the vesicles, stem cell activity as measured by EdU incorporation, and the formation of BC. As shown in **Figure 6**, both SB431542 and A8301 did not induce damage to the vesicles, nor did they significantly affect stem cell activity. The formation of BC, however, was highly significantly diminished by both inhibitors. The blocking of BC formation by this pharmacological treatment was, thus, not due to general toxic effects of the inhibitors on *Echinococcus* stem cell proliferation but specifically affected the differentiation towards anterior identities within the GL.

Since both human BMP2 and TGFβ were able to stimulate *Echinococcus* TGFβ/BMP receptors in the HEK293 T cell system, we finally also tested the effects of these cytokines on *Echinococcus* brood capsule formation. Again, we used vesicles of isolate MB17 at a size of 5 mm and added both cytokines at a final concentration of 1 nM. As shown in **Figure 6**, BC developed in metacestode vesicles under all conditions. However, in the presence of BMP2 and TGFβ, BC formation started earlier and, at a time point after two weeks, significantly more vesicles contained BC when treated with BMP2 or TGFβ.

Taken together, these analyzes demonstrated that *E. multilocularis* metacestode vesicles predominantly formed BC and protoscoleces when cultured aerobically with host cells and that BC formation is specifically inhibited in the presence of compounds that target EmTR3 and EmTR4. Furthermore, human cytokines which stimulated the parasite TGFβ/BMP receptors in the heterologous HEK293 T expression system, also accelerated BC formation *in vitro*. These data indicated that functional TGFβ/BMP signalling is important for BC formation by metacestode vesicles.

### Co-cultured metacestode vesicles express higher levels of parasite activin

**Figure 7:**
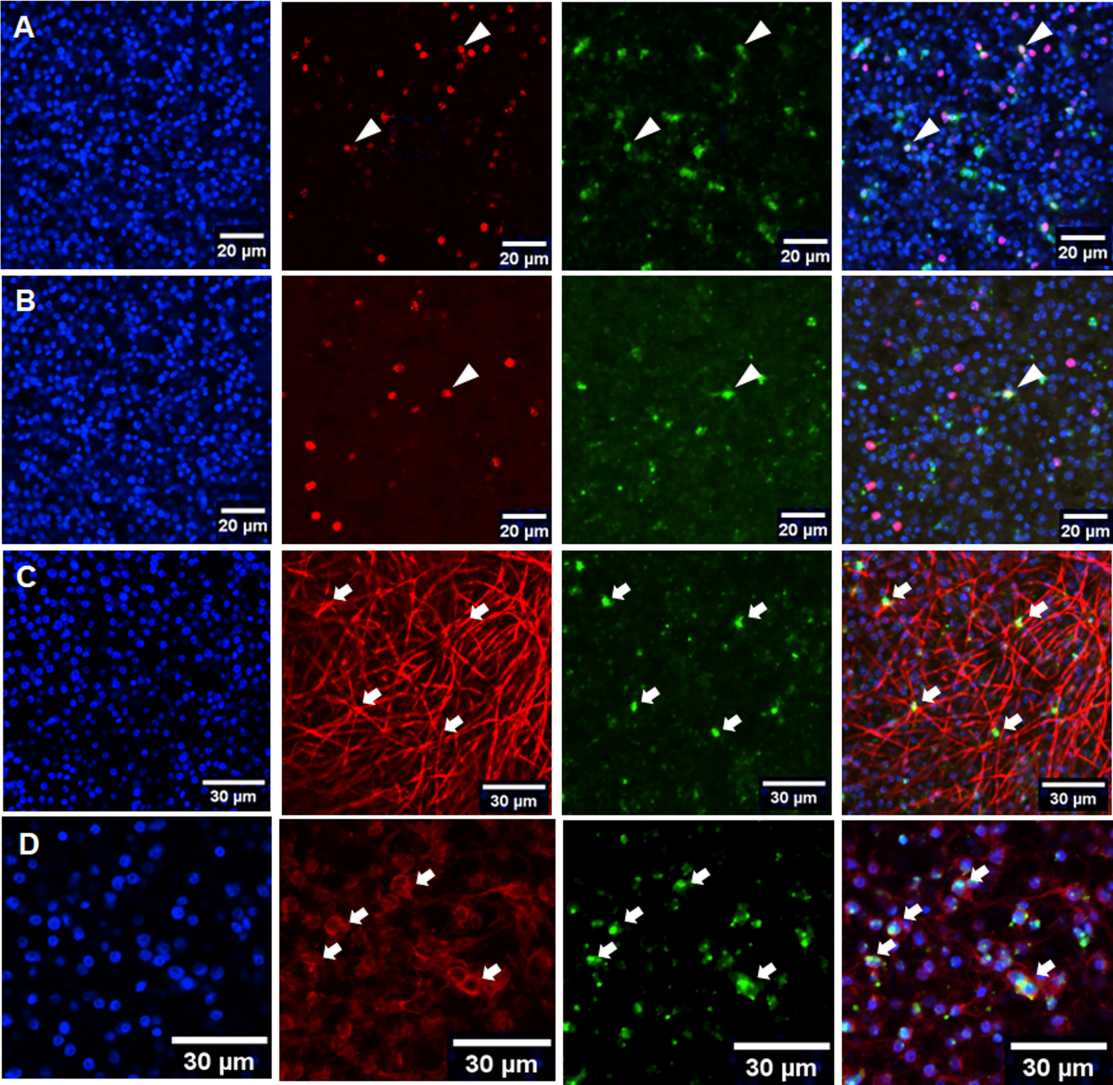
Expression of *Emact* within the metacestode GL. Representative WISH images are shown for *Emact* expression in the GL of axenically cultivated vesicles **(A)** and vesicles from co-culture **(B)** as well as in muscle **(C)** and nerve cells **(D)** from co-cultured vesicles. Channels indicate DAPI (nuclei, blue), *Emact*-specific probe (green), EdU (S-phase stem cells, red; **(A, B)**), anti-HMW tropomyosin (muscle fibers, red; **(C)**), and anti-AcTub (nerve cells, red; **(D)**). Merges of all three channels are shown in right panel. Cells double positive for *Emact* and EdU are indicated by white triangles **(A, B)**, double positive for HMW tropomyosin and *Emact* by white arrows in **(C)**, and double positive for *Emact* and AcTub by white arows in **(D)** Bar indicates 20 µm in **(A, B)** and 30 µm in **(C, D)**.

Since only vesicles from co-culture formed BC, we assumed that the feeder cells either produced stimulating cytokines for BC development and/or that the parasite vesicles themselves were in a state that facilitated BC formation. To investigate this aspect, we performed RT-qPCR experiments on vesicles from co-culture and for axenically cultivated vesicles for all four parasite TGFβ/BMP receptors and for the previously characterized parasite Smads. Furthermore, we included in these analyzes a gene encoding an activin-like parasite ligand, EmACT, which we had previously identified to be secreted by *Echinococcus* larvae and which induced Foxp3+ Treg conversion, probably by stimulating host TGFβ receptors (Nono et al., 2020). As shown in **Figure S4**, there was no significant difference in the expression of genes encoding R-Smads and the Co-Smad, EmSmadD, in vesicles under both culture conditions. Likewise, no differences were observed for *Emtr1* and *Emtr2* under both culture conditions (**Figure S4**). For *Emtr3* and *Emtr4*, however, expression levels in co-culture were significantly higher than in axenically cultured vesicles (2.5- to 3-fold). Interestingly, we also observed a markedly higher expression of the EmACT encoding gene, *Emact*, in co-culture vesicles than in axenically cultured metacestodes. To investigate whether this was due to a higher number of *Emact* expressing cells in co-cultured vesicles, we performed *Emact*-specific WISH on vesicles from co-culture and from axenic culture (**Figure 7**). In both cases, however, we found similar numbers of *Emact* expressing cells (7 ± 2% of all cells for axenic culture, n = 1755; 8 ± 2% for co-culture, n = 2115) within the germinative layer, indicating that higher expression levels of *Emact* per cell account for the higher levels of gene expression observed in RT-qPCR experiments instead of higher numbers of *Emact* expressing cells within the GL. Taken together, the RT-qPCR studies indicated that components of the TGFβ/activin branch *Echinococcus* are significantly upregulated in co-culture conditions.

### *Emact* is predominantly expressed during protoscolex formation

We were further interested in the cells expressing *Emact*, and in the role of this gene in brood capsule formation and, thus, performed *Emact*-specific WISH. As shown in **Figure 7**, *Emact* was expressed throughout the germinative layer, predominantly in post-mitotic cells. In rare cases we also observed *Emact* expression in S-phase stem cells (less than 1% of all cells in both co-culture and axenic culture). Since the vast majority of *Emact* expressing cells were post-mitotic, we then performed *Emact*-specific WISH combined with staining for HMW tropomyosin (muscle cells) and staining against AcTub (nerve cells). As shown in **Figure 7**, we observed *Emact* signals in numerous cells associated with muscle fibers, although, as already outlined above, exact quantification is hard to achieve due to difficulties in precisely assigning muscle cell bodies to the respective fibers. The majority of *Emact*+ cells was found associated with AcTub of which 80-90% stained positive for *Emact* (**Figure 7**). Taken together, these data indicated that *Emact* is expressed in very few S-phase stem cells, whereas most of the cells expressing the parasite cytokine are post-mitotic cells, including muscle and nerve cells.

**Figure 8:**
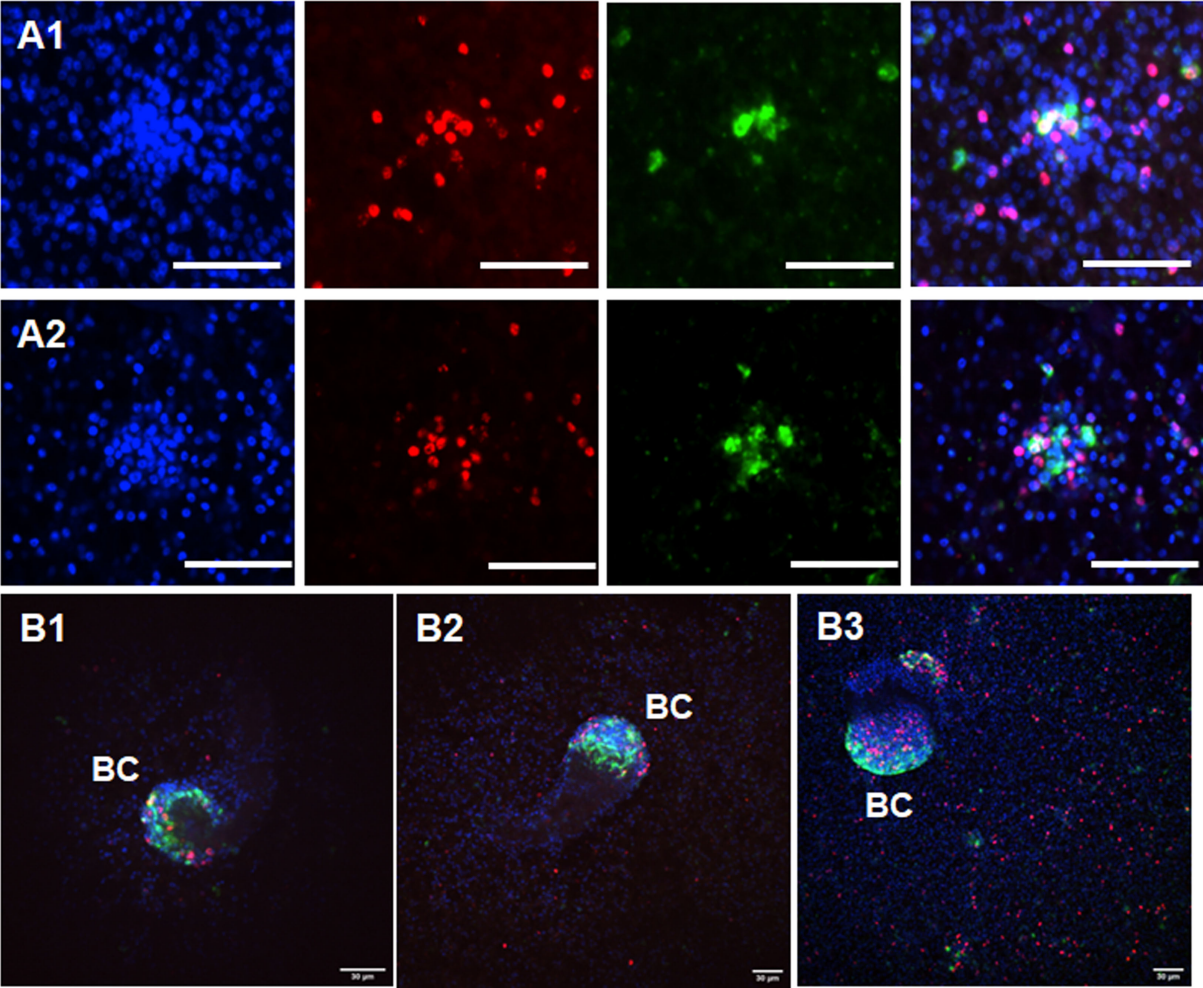
Expression of *Emact* in BC. Two examples of representative WISH images are shown for *Emact* expression in early BC in **(A1, A2)**. Shown from left to right are DAPI channel (nuclei, blue), EdU (S-phase stem cells, red), *Emact* (green), and merge of all three channels. Bar indicates 30 µm. **(B1–B3)** shows three examples of BC in different stages of development. Channels are as in **(A1, A2)**, shown are merge images of all three channels. BC indicates brood capsules. Bar represents 30 µm.

**Figure 9:**
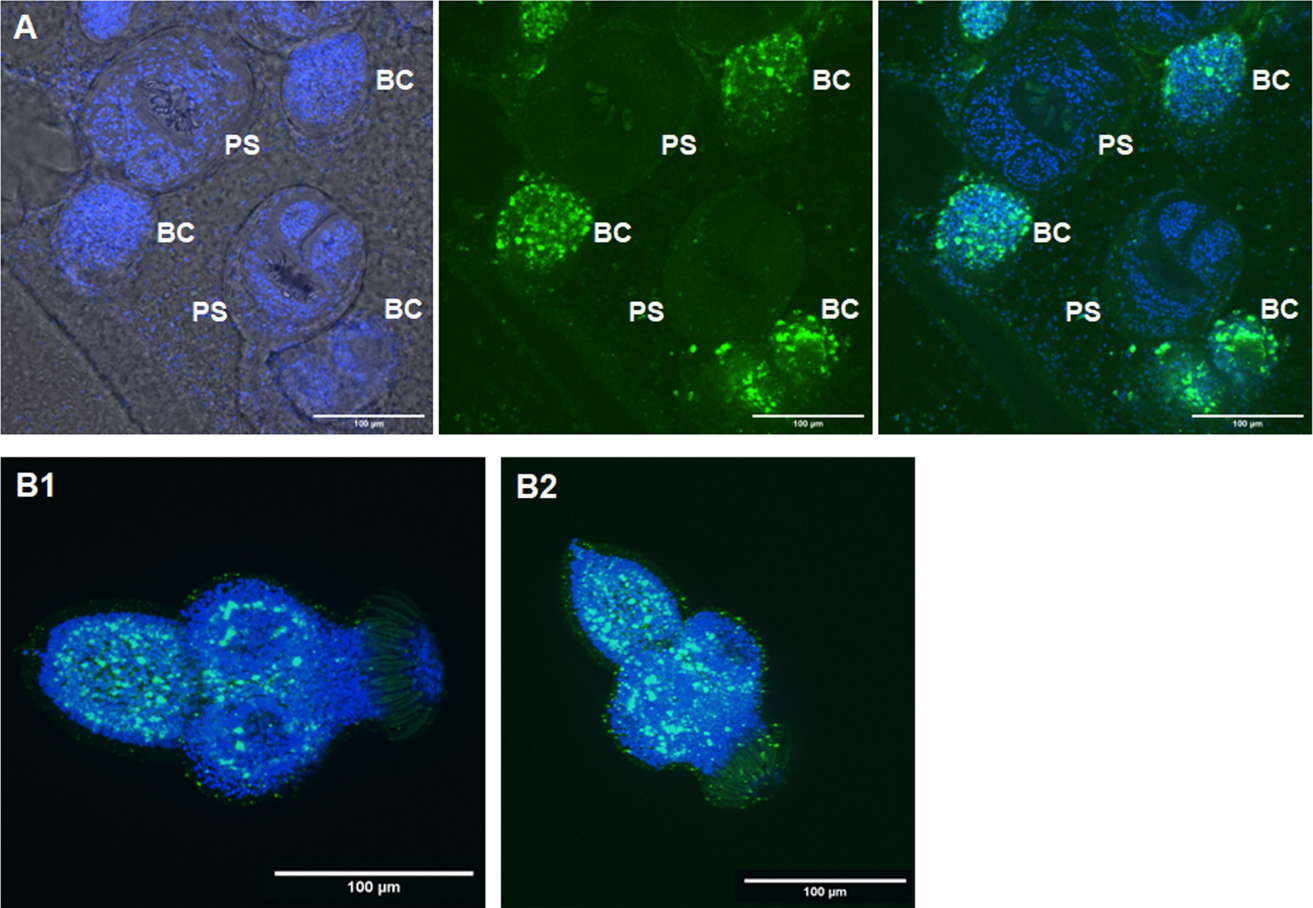
Expression of *Emact* in protoscoleces. **(A)** shows a representative WISH analysis of *Emact* expression in dormant protoscoleces (PS). Left channels is DAPI (nuclei, blue), middle channel is *Emact* signal (green), right is merge of both channels. Bar represents 100 µm. Please note strong *Emact* signals in BC immediately adjacent to dormant PS. **(B1, B2)**, two examples of representative WISH images for *Emact* expression in activated PS. Displayed are ImageJ 3D projects of 20 stacks. DAPI (nuclei, blue) and *Emact* (green) are merged. Bar represents 100 µm.

We previously identified *sfrp*, a canonical marker for the anterior pole, as one of the earliest genes expressed in BC (Koziol et al., 2016). In the earliest developmental stage, BC can be identified as an accumulation of cells within the GL with locally elevated numbers of EdU+ cells, indicating increased proliferative activity (Koziol et al., 2014). To investigate a possible role of *Emact* in BC formation, we therefore cultivated vesicles of isolate MB17 for two weeks after having reached a size of 5 mm in diameter, still before BC were visible by light microscopy. The vesicles were then subjected to a 5 h EdU pulse, fixed, and analyzed for *Emact* expression following WISH. In the DAPI channel we first screed these vesicles for cell accumulations and then added the EdU channel to identify potential BC in early development (see **Figure S5**). Interestingly, around 80% of the structures thus identified also showed intense *Emact* signals, indicating that *Emact* is one of the earliest genes expressed in BC at the onset of protoscolex formation (**Figure 8**). We then analyzed vesicles with very small but clearly visible BC (as assessed by light microscopy) and found strong *Emact* signals always associated with the most anterior domain during protoscolex formation (**Figure 8**). Interestingly, no *Emact* signals were obtained for dormant forms after completion of protoscolex formation, although developing BC adjacent to these protoscoleces still displayed a high number of *Emact*+ cells (**Figure 9**). Finally, we also analyzed pepsin/low pH activated protoscoleces for *Emact* expression and found the gene expressed in anterior as well as posterior parts (**Figure 9**) in a pattern resembling the protoscolex nervous system (Koziol et al., 2013).

Taken together, these analyzes indicated that *Emact* is expressed throughout the parasite’s GL during infiltrative growth of the vesicles before BC formation, predominantly in nerve and muscle cells. *Emact* then shows strong expression in early BC and is subsequently expressed in the anterior BC domain until protoscolex formation is completed. In the dormant protoscolex, *Emact* expression ceases until, upon activation of protoscoleces during the infection of the definitive host, *Emact* expression is reactivated.

### BC formation is induced by supernatant from BC forming vesicles

**Figure 10:**
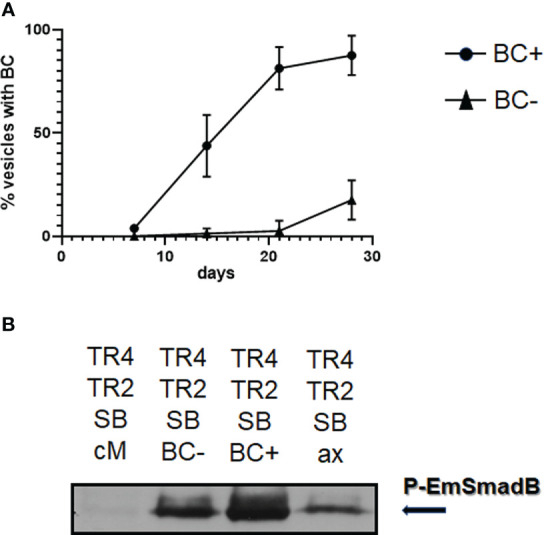
Induction of BC formation by culture supernatant. **(A)** Influence of supernatant from BC containing and BC free vesicles on BC formation. Metacestode vesicles of isolate MP17 were grown for 28 days in co-culture in the presence of conditioned medium obtained from cultures with BC containing vesicles (BC+) or BC free vesicles (BC-). BC formation was assessed light microscopically. Shown are median with standard deviation. Unpaired t-test as assessed by GraphPad prism revealed (p < 0.0001) at day 14 between BC+ and BC-. **(B)** Influence of culture supernatant on the activity of *Echinococcus* TGFβ/BMP receptors. The *E*. *multilocularis* receptors EmTR4 and EmTR2 were co-expressed with EmSmadB in HEK 293 T cells and exposed to supernantant from cultures with BC containing vesicles (BC+), BC free vesicles (BC-), or from axenically cultured vesicles (ax). Conditioned medium (cM) was used as a control. Cell lysates were subjected to PAGE and Western blot analysis using an antibody against phosphorylated EmSmadB. Arrow to the right indicates the phosphorylated R-Smad forms. Comparative amounts of total R-Smad, as measured using an anti-Myc-tag antibody, were applied to the gel. Result shows representative example of experiment performed in triplicates.

During our investigations, we had made two important observations concerning BC formation: First, vesicles did not show gradual BC formation but, upon the onset of BC formation in certain regions of the vesicle, developed microscopically visible capsules over the entire vesicles within 1 - 2 days. Second, once a vesicle within a culture well started developing BC, the remaining vesicles of the well followed soon (5 - 7 days) after. Hence, BC formation in one region of metacestode tissue appeared to induce the onset of BC development in adjacent regions and even in other vesicles which were cultured in the same well. To further investigate this aspect, we set up vesicle cultures of isolate MB17 of 5 mm in diameter and incubated these with supernatant from MB17 vesicles without or with developing BC and followed BC formation over 28 days. As can be seen in **Figure 10**, vesicles incubated in supernatant of BC containing vesicles developed BC much earlier than those incubated with supernatant from BC free vesicles, indicating that *Echinococcus* BC formation indeed positively influences BC formation in neighboring vesicles. To closer investigate the molecular nature of the BC inducing molecules in these experiments, we tested the ability of the respective supernatant for inducing the activity of EmTR2/EmTR4 in the HEK293 T cell system. As shown in **Figure 10**, supernatant from rat Reuber feeder cells alone did not stimulate the phosphorylation of EmSmadB by EmTR2/EmTR4 whereas a clear signal was obtained for supernatant from *Echinococcus* co-cultures in which the vesicles were BC free. The strongest signal was obtained for supernatant of cultures from BC containing vesicles (**Figure 10**). A faint signal was obtained for supernatant from axenically cultured vesicles.

Taken together, these data indicated that *Echinococcus* metacestode vesicles release a factor (or factors) capable of inducing BC formation in other vesicles and a factor (or factors) that stimulate the activity of EmTR2/EmTR4.

## Discussion

As metazoans, parasitic helminths are phylogenetically relatively closely related to their mammalian hosts with which they share numerous cell-cell communication systems. During recent years, this has led to the concept of host-parasite cross-communication involving evolutionarily conserved signalling systems for which several examples have been described so far. In the case of *Echinococcus*, we had previously established that host hormones and cytokines such as insulin or fibroblast growth factor can directly stimulate respective receptor tyrosine kinases expressed by the parasite, thus stimulating growth and proliferation of the *E. multilocularis* metacestode (Hemer et al., 2014; Förster et al., 2019). Likewise, as demonstrated by Cheng et al. (2017), mammalian epidermal growth factor can stimulate a cognate receptor kinase expressed by the *Echinococus* metacestode, leading to enhanced stem cell proliferation. In the present study, we extend the list of *Echinococcus* receptors capable of responding to host cytokines to the family of TGFβ/BMP receptors. We demonstrate that mammalian BMP2 stimulates EmTR1 and EmTR4 when co-expressed with the type II receptor EmTR2, which results in the phosphorylation of downstream R-Smads, clearly indicating that the respective *Echinococcus* receptors are functionally active kinases. In the case of EmTR4/EmTR2 we also showed that physiological concentrations of TGFβ are recognized by parasite receptors, which is particularly interesting since it is already known that elevated concentrations of TGFβ are accumulating around parasite lesions in late stages of AE (Wang et al., 2013). Our data are in line with previous studies on related flatworm receptors demonstrating interaction with host TGFβ/BMP cytokines. In the case of the related cestode *Taenia crassiceps*, Adalid-Peralta et al. (2017) showed that human TGFβ at elevated concentrations prevents binding of specific antibodies to orthologs of EmTR1 and EmTR2. In contrast to the work of Adalid-Peralta et al. (2017), however, our study demonstrated that host TGFβ not only binds to cestode TGFβ/BMP receptors but also induces receptor activation and phosphorylation of downstream R-Smads. In the related trematode *S. mansoni*, Beall and Pearce (2001) showed that human TGFβ both binds to and activates a close ortholog of EmTR3. It is thus highly likely that functional interactions with host derived TGFβ/BMP cytokines are a general feature of all flatworm TGFβ/BMP receptors, particularly since we herein showed that also mammalian BMP stimulates respective receptor activities. Although EmTR3 in the heterologous HEK293 T cell expression system did not require the addition of host cytokines for full activation (**Figure 3**), we propose that even this receptor is responsive to host TGFβ since (i) this is also the case of the schistosome ortholog SmRK1 (Beall and Pearce, 2001) and (ii) in heterologous type I/type II receptor complexes, TGFβ usually first binds to the type II receptor component, which then recruits the type I receptor for full activation (Johnson and Newfeld, 2002; Massagué, 2020). Hence, in our experimental setting we assume that traces of TGFβ present in serum and/or released by HEK293 T cells were already acting on the recombinantly expressed parasite receptors. Expression of TGFβ by HEK293 T cells in culture has already been shown previously (Jazi et al., 2016). Taken together, the analyzes presented herein not only characterized the full TGFβ/BMP receptor complement of *E. multilocularis* and showed that these molecules are functionally active, but they also demonstrate that different combinations of EmTR2 and parasite type I receptors are responsive to host BMP2 and/or TGFβ. Whether this is also the case for EmTRL, which contains an extracellular region typical of TGFβ/BMP receptors, remains to be established as are questions concerning possible activities of this molecule as a pseudokinase (Byrne et al., 2017). Although EmTRL did not show significant overall homologies to the well-known pseudoreceptor BAMBI, which modifies TGFβ signalling in humans (Onichtchouk et al., 1999), a similar function in *Echinococcus* cannot be excluded.

Our inhibitor studies indicated that functional TGFβ/activin signalling is not crucial for proliferation and survival of the metacestode but is necessary for proper BC and protoscolex formation. Since TGFβ/activin receptors are usually activated in response to cognate cytokine stimuli, this raises the important question whether BC formation is induced and/or supported by host cytokines such as BMP2 or TGFβ, or whether the parasite itself provides respective cytokines. Since BC formation is regularly induced in metacestode tissue during co-cultivation with rat Reuber hepatoma cells (**Figure 6**) but those feeder cells alone apparently do not secrete molecules that activate parasite TGFβ receptors (**Figure 10**), we rather propose that one major stimulus for BC induction derives from the parasite. This is supported by our data showing that culture supernatant of BC containing vesicles can induce BC formation in other vesicles and that such supernatant stimulates the activity of EmTR4/EmTR2 when heterologously expressed in HEK293 T cells, particularly in the case of BC developing cultures (**Figure 10**). A likely candidate for the BC inducing cytokine is EmACT, the expression of which strongly correlates with BC inducing activities of respective cultures supernatants. As we have shown, the EmACT encoding gene, *Emact*, is expressed throughout the GL in BC free vesicles, is strongly induced during BC formation, and shows lower expression in vesicles from axenic culture, which do not form BC. We thus propose that EmACT by stimulating EmTR2/EmTR4 and most probably also by acting on EmTR3/EmTR2 is an important parasite-intrinsic factor that induces BC formation, which may be supported in its activity by host derived cytokines such as BMP2 and TGFβ. At least in the co-culture system, addition of these cytokines to metacestode vesicles accelerated BC formation, even in the presence of EmACT (**Figure 6**). We cannot rule out at present that apart from TGFβ/activin-like ligands from host and parasite, also BMPs might have a role in *Echinococcus* BC formation, particularly since mammalian BMP2 both stimulated the activity of EmTR4/EmTR2 (**Figure 3**) and accelerated BC formation when added to the co-culture system (**Figure 6**). Indeed, as we have reported previously (Brehm and Koziol, 2017), the parasite genome encodes two BMP-like ligands, EmBMP1 and EmBMP2, which are expressed in the metacestode according to transcriptome data (Tsai et al., 2013). However, when we performed WISH on both genes, we never obtained clear signals. Furthermore, when using the BMP-branch-specific inhibitor Dorsomorphin on parasite cultures, BC formation was not inhibited (data not shown). Lastly, and unlike TGFβ, there are no indications of elevated host BMP concentrations around parasite lesions during AE *in vivo*. Hence, cytokines of the BMP branch appear to have much less impact on *Echinococcus* BC formation and development than those of the TGFβ/activin branch.

As we have previously established, the *E. multilocularis* metacestode is a highly unusual developmental stage that results from a modification of the parasite’s body axes (Koziol et al., 2016). According to these studies, *E. multilocularis* utilizes gradients of anteriorizing and posteriorizing morphogens such as sFRP or Wnt-1, respectively, to establish the anterio-posterior body axis in the protoscolex (Koziol et al., 2016). At least for the related tapeworm *Hymenolepis microstoma*, we also showed that the anterio-posterior body axis of the oncosphere is defined by opposing *sfrp* (anterior) and *wnt1* (posterior) expression (Koziol et al., 2016), indicating that these genes most likely also define the AP axis during the development of *Echinococcus* oncospheres. Either at the end of oncosphere development or during the oncosphere-metacestode transition, the anterior pole appears to be suppressed, resulting in the strongly posteriorized metacestode, which expresses *wnt-1* over the entire GL and which lacks any anterior identities (Koziol et al., 2016). The chronic phase of AE is then characterized by almost unrestricted, infiltrative growth of the metacestode within host tissue until, at the end of the infection, the anterior pole (i.e. *sfrp* expression) is established again at numerous sites within the GL, resulting in the production of protoscoleces (Koziol et al., 2016; Koziol, 2017). In the related but free-living planarians, body axis formation is crucially regulated by position control genes (PCG), the majority of which is expressed in muscle cells (Witchley et al., 2013). Accordingly, we previously demonstrated muscle-specific expression of some of those genes (e.g. *wnt-1*, *wnt-11a*, *sfrp*) in the *Echinococus* metacestode (Koziol et al., 2016), which also explained why the non-motile metacestode contains muscle cells. Although the planarian activin orthologs (*activin-1*, *activin-2*) do not, *per se*, belong to the group of PCG, a crucial role of *activin-2* in head regeneration has already been firmly established (Gaviño et al., 2013; Roberts-Galbraith and Newmark, 2013) and Cloutier et al. (2021) recently demonstrated that activin signalling is important for the regeneration of polarity on the planarian anterior-posterior axis. Interestingly, as with planarian PCG, *activin-2* is mainly expressed in planarian muscle cells (Scimone et al., 2018), whereas the planarian activin receptor (*ActR-1*) and the cognate R-Smad (smad2/3) are broadly expressed, but neurally enriched (Roberts-Galbraith and Newmark, 2013). Likewise, single-cell sequencing in schistosomes revealed muscle- and nerve cells as the major cell types expressing TGFβ/BMP signalling components (Wendt et al., 2020). Our data are in line with these findings showing that *Emact* and the *Echinococcus* TGFβ/BMP receptor encoding genes are predominantly expressed in neurons and in muscle cells of the metacestode whereas only very few germinative (stem) cells showed positive signals for these components. Since in *Echinococcus* only the stem cells are capable of proliferation and, thus, of protoscolex and BC formation (Koziol et al., 2014), the situation appears largely similar as in planarians where muscle cells and the nervous system provide the coordinate system for body axis formation to which the stem cells respond by producing differentiated progeny to precisely replace missing body parts (Witchley et al., 2013). The muscle and nerve cell-specific production of stimuli for BC formation also explains why metacestode vesicles are only capable of protoscolex production when they have grown to a certain size since both the metacestode muscle and nerve cell systems are not yet fully developed in very small (young) vesicles (Koziol et al., 2013).

Although we observed a strong correlation between the appearance of early BC and *Emact* expression, we cannot yet tell whether *Emact* acts upstream of *sfrp* in determining the anterior pole or whether *Emact* expressing cells are formed (or recruited) after *sfrp* expression (i.e. as a downstream mediator of BC development). Respective investigations would require the establishment of protocols for double WISH, which are currently not available for *Echinococcus*. During planarian regeneration, re-establishment of the anterior pole involves distinct cells which co-express the *wnt* and activin signalling inhibitors *notum* and *follistatin* (so-called anterior pole cells), respectively, and which later give rise to *sfrp* expressing cells (Vogg et al., 2014). The role of TGFβ/activin signalling in this process appears rather complex since single RNAi knockdown of activins or activin receptors in planarians does not *per se* result in dramatic phenotypes concerning the formation of the anterior pole (Gaviño et al., 2013; Roberts-Galbraith and Newmark, 2013). The head regeneration defective phenotype of *follistatin*(RNAi) planarians does, however, require functional activin signalling (Gaviño et al., 2013; Roberts-Galbraith and Newmark, 2013) and one important role of activin in planarians is asymmetric expression of *notum* in muscle cells at wound sites (Cloutier et al., 2021). Hence, at least during planarian regeneration, the formation of the anterior pole is governed by a complex interplay of posterior *wnt* and anteriorizing TGFβ/activin agonists and antagonists within muscle and nerve cells (Sureda-Gomez and Adell, 2019). In *Echinococcus*, orthologs to *follistatin* and *notum* have not yet been characterized, although both factors are encoded in the genome and, according to RNA-Seq data, are also expressed in the metacestode (Tsai et al., 2013). Clearly, further investigations into the complex interplay between *wnt-1*, *Emact*, *follistatin*, and *notum* are necessary to elucidate the complex events leading to re-establishment of the *Echinococcus* anterior pole within the GL. Respective studies should also include possible interactions between parasite *follistatin* and human TGFβ since these could influence the relative dynamics of BC formation induced by parasite versus host factors. Anyhow, based on our findings that (i) host TGFβ accelerates BC formation in metacestode vesicles, that (ii) inhibition of *Echinococcus* TGFβ/receptors prevents BC formation, and that (iii) supernatant of BC containing vesicles stimulates both BC formation in vesicles and parasite TGFβ receptor activity, we favor a model in which *Echinococcus* TGFβ/activin signalling generally counteracts the posteriorizing effects of *wnt* signalling in the metacestode. We suggest that BC formation is suppressed in the *wnt* high environment of the metacestode so long as TGFβ/activin ligands are only moderately expressed, and that the accumulation of such ligands around parasite lesions at later stages of the infection, maybe through localized *wnt* inhibition, skew the balance in favor of anteriorizing structures, eventually leading to BC formation.

In *E. multilocularis*, the process of BC formation and protoscolex development must be finely tuned. The delay of anterior pole formation, obtained through posteriorization of the metacestode, favors unrestricted spreading of metacestode tissue within host organs, which later results in the production of maximized numbers of protoscoleces. Since metacestode vesicles cease to grow once BC formation is induced, premature establishment of the anterior pole would result in less metacestode mass and, thus, reduced numbers of protoscoleces to be passed on to the definitive host. On the other hand, if the intermediate host is taken by the definitive host when protoscoleces have not yet formed (i. e. too late), the infection of the intermediate host would have been a dead end for the parasite. For *E. multilocularis* it would thus be advantageous to ‘sense’ the end point of the infection process. We suggest that the increasing suppression of the host immune response by the metacestode, which is in part induced by EmACT (Nono et al., 2020) and which leads to the accumulation of TGFβ secreting Foxp3+ T-reg around the parasite (Wang et al., 2013), may serve as an important clue for determining this end point. In natural intermediate hosts, this scenario would lead to maximized numbers of protoscoleces that are passed on to the definitive host. Since humans are generally considered relatively resistant to AE (Vuitton and Gottstein, 2010; Gottstein et al., 2015) and accumulate less TGFβ around parasitic lesions (Wang et al., 2013), it would also explain why human infections are rarely associated with protoscolex formation.

## Data availability statement

The datasets presented in this study can be found in online repositories. The names of the repository/repositories and accession number(s) can be found in the article/**Supplementary Material**.

## Ethics statement

This study was performed in strict accordance with German (Deutsches Tierschutzgesetz, TierSchG, version from Dec-9-2010) and European (European directive 2010/63/EU) regulation on the protection of animals. The protocol was approved by the Ethics Committee of the Government of Lower Franconia under permit numbers 55.2–2531.01-61/13 and 55.2.2-2532-2-1479-8.

## Author contributions

KB, MS, and UK conceived and designed the experiments. MK, KE, PB, KR, IT, NL, MB, and MS performed the experiments. MK, KE, PB, KR, IT, NL, MB, MS, UK, and KB analyzed the data. KB wrote the manuscript. All authors contributed to the article and approved the submitted version.
