# Supplementary material for: Transforming growth factor-β signalling regulates protoscolex formation in the Echinococcus multilocularis metacestode
Source: Front Cell Infect Microbiol. 2023 Mar 22;13:1153117. doi: 10.3389/fcimb.2023.1153117 (PMC10073696; doi:10.3389/fcimb.2023.1153117)
Supplement: Supplementary file 1 [file DataSheet_1.pdf]

Fig. S1

```
EmTRL     ------------------------------------------------------------     0
EmTR2     -------------------MEQISCYCTPDQPGCNPITRKIL------------------     23
EmTR3     MFALSLLLFSAAKPSIANRKECLKLLCNPDESDCHPCAGMSVDELGTWRRYIDALLQNFN     60
EmTR1     -----------------MK-----------------------------------------     2
EmTR4     -----------------MR-----------------------------------------     2

EmTRL     ----------MTWRGSLVCVCNDNMHCFQNKTLNSMFNLPMEIRFCIAPFRGFCYASPHK     50
EmTR2     ------------------CKS-GQQHCLLSLKQLDD------HSNR--------------     44
EmTR3     DEESSNAYYRQIWEEPFCCTISPGERCIIK---LI-------------------------     92
EmTR1     -------------KHTILCKCFPSETCVER------------SSEC--------------     23
EmTR4     -------------HKIINCFCYPFERCDSS---GPS------NSTC--------------     26
                            *       *

EmTRL     THELPLYLQDSYWSAPKVDTYSASLEGFSQDVSFKYGCLHFSPFLFFMCSGHPLDRGANT     110
EmTR2     -----THLQGCW--------YWRSN---------------NDFVFESN--VCYVVSNKKG     74
EmTR3     -----------------------NEPNVPPHRLYEMGCLRQKDFWKPF--SCE-----SA     122
EmTR1     -----ASLIGCF--------YSVQKDALGYVVDEHYGCLLNNSFSIIS---CL--NFRGT     65
EmTR4     -----VTTTGCF--------TARNII--NGTEYITSGCFSNDIFQMQI--TCGVLSNKNS     69
                                    .               . .

EmTRL     LLSCCSKNNTCNYYLRFRSPYTKTFDSITGNEKVHKQNRDLGKPSFEHMSLEAMPFMSDT     170
EmTR2     YVTCCCKGDSCNVPHIPIKQIVIDQGDP---THSAPFNRSLGN----HISTTKIGVLAS-     126
EmTR3     NMRCC-NSSFCNLPLKEELS------------------------------K----LIVE-     146
EmTR1     NTTCCFSSNSSDYC---NAHLPVNKQ----------------------------------     88
EmTR4     SIYCCSNGNFCNWSNFSDLFLPFLSKSQ---YSVSSY-ASLSR----NIRT----AMYD-     116
             ** ... .:

EmTRL     PFGPELLMKSHSLLKSDKHLTEPHEQGTTSPGLISVKRLPPYIPMIILAGVIFLLVIIRF     230
EmTR2     -----------------------------------FSSNPTLSYAVALPL-L--LLVL-L     147
EmTR3     -----------------------------------VPKDNTLI--VILSI-FLVLAVLML     168
EmTR1     ------------------------------------NTGALFF-P--LTF-FTVLCILLF     108
EmTR4     -----------------------------------WSSEAILI-SLISSV-IVFFCILAF     139
                                                             :  : :: :

EmTRL     VSFLLFSNYFHQKRKESE--------------------------------------RCSW     252
EmTR2     IFIFIFLLCRRCK--------TSAHPL-GSSAF----GGSSKFSTDK---------RKSL     185
EmTR3     VAGWCFWRCRDKADWRKS--SQAPVSMIGAGCIGDATGHTSPSDTHPWHMAECGASGASM     226
EmTR1     VLA---FVYI--------------------------------------------------     115
EmTR4     FLF---FICFSRKGLVTRPCRCKQLPYTGAVHCSDRNRNVGKFLTKPFFMKHDLNICSSM     196
          .

EmTRL     VCCITSPKPK-----LVLNSSSVAST-------NST------------------------     276
EmTR2     FCRNPFPTSLKFC----FSTSRSK---------------------DDEHQFC-A------     213
EmTR3     VSAIPTATTTANASAVAVNGGVLNATAANSGVSVATTLIDSVPLHPSRTPFLMASSSISS     286
EmTR1     ----------------------------------------KGNFSKHEK-----------     124
EmTR4     HCPY----SLMCCSKICINTS-LNLT--------QTNLLKNNPFSNHVL-----------     232

EmTRL     ------------------------------------NLQESQSLF---------------     285
EmTR2     ------------------------SGNIVCRS---CPLNGAPCVFCGKGFLIQ-VDGSSS     245
EmTR3     VIAVRPPRGLGAIPTGVSSYHNSIGGSTVVRASEVVGLGGGGA-----SYTAA-SGGASM     340
EmTR1     ------------------------------------PLTK-H------------------     129
EmTR4     ------------------------------------QLTN-SS-----SFPQKSLESAAA     250
                                               *
                                                           G
EmTRL     --------------------SSQFIQNKTFY--GQMVAHKLEFLTSFSRDRDSEILSAKY     323
EmTR2     MPR---------------LVRPVLGGGWPAEGEIEELSRICTKVKRCSRGRFGEVWLGRM     290
EmTR3     LPTSCSAPPGAGGTLEITLSGSGSGAGQPLLV-ERTVARQVTLSARIGEGRYGEVWLGRL     399
EmTR1     --S---------SPYFPEFTDSGSGSGKPFLV-SQTIARQTTLLVCIGKGRFGEVWRAVC     177
EmTR4     LPS---------ETQPSGTMSSGSGSGVPFLV-QATIARQISLQECIGKGRYGEVWRGIY     300
                                    .         :::        ...* .*:  .
                         K                E
EmTRL     K--------DEVVSVRLLHPTSPSRSLMLWKRLTCLHEKCVLRHSSLSGINAADVCLLAD     375
EmTR2     TEVVSDLPTSREVAIKV----FPEAEKKSWETELELYRLPRLKHPNILHYIGIDKVTRVL     346
EmTR3     H--------GDQVAVKI----FSSRNENSWIREKEIYETATLRHSNILGFIAADNKD---     444
EmTR1     N--------GEVVAVKI----FSSRDGASWTRETQIYTTALLSHPNILAYYASDMIS---     222
EmTR4     R--------GENVAVKI----FSSRDEASWARETHIYSSVLLRHENILSYYASDITS---     345
                   .  *::::      . .   *     ::    * * .:    . *
```

```
EmTRL      LRCHAMPHSTSEPTATSALCAFTISEFYPWGSLFNFMQQNSPTDSQYTSSALTFTLCALI      435
EmTR2      SEEGENDQNSGVGEGGTVAEYWLVTDYLAFGSVFDYIHSRE-LSWG-------QMLWVAM      398
EmTR3      --------------NGISTELWLIAEYHRLGSLYEFLQSHA-FTLP-------ALIKMAS      482
EmTR1      --------------RGGCTQLWLVTAYHAAGSLHDFLSTAKGVTPQ-------CGLKLAR      261
EmTR4      --------------RYGCTQLWIISHYHPYGSLYDFLQAH-VVDTI-------AALRLAK      383
                                : :: :   **:.:::                  :
                                       D    N            D
EmTRL      DVVNGITFLHSDMA---GTRGRPALAHRNICLENLHLKVDGGICIGGLEYAICAPPSPLP      492
EmTR2      GMARGLSYLHTELPRTVSQYAKPSIAHRDFKSRNVLLKPDLTPCISDLGLATRLETGRG-      457
EmTR3      SIANGLAHLHMAIV---GTSGKPPIAHRDLKSRNILVKADGECCIGDLGFAVKYDSLTG-      538
EmTR1      SIAAGLAFLHSEVV---GFHGKPPIAHRDIKSKNILVMANNEACLADFGLALVKTSKGMN      318
EmTR4      TAASGLCYLHSYIL---GLQGKPAIVHRDIKSKNILVQANGTCSIGDPGLAVMQAPGST-      439
             . *: .**  :    .  .:* :.**::  .*: :  :   .:..   *

EmTRL      LPAEQLMQIFNAHLRDWSAAQLPIYSEFHCSNVFSDSDHRAPIASVPLYPDWWPVCGLQV      552
EmTR2      ----------------------------------------------------FGDSHLQV      465
EmTR3      -------------------------------------------------RVDIGHNTERI      549
EmTR1      GGGT------------------------------SDE--------ANESGDALPPASLFA      340
EmTR4      -------------------------------------------------EMDVGQPNHMV      450


EmTRL      GLPTYMAPEIMEETL---------------------------------------------      567
EmTR2      GTARYMAPEVLDGAI---------------------------------------------      480
EmTR3      GTKRYMAPEVLNNTL---------------------------------------------      564
EmTR1      GTKRYMAPEILALYPLVW-----GGWVRARTQERQIDKKQSGECDEDNLSIPGELLECRH      395
EmTR4      GTKRYMAPEILADDGLEECPESGEGFLPHDFEISAYL--QS------KNRINDTSPVLNT      502
           *   *****::

EmTRL      FPFCFESHKRADVYALGIVIWEIATWALGQSVGHF-----------WVP------RDADI      610
EmTR2      -QFTRDAFLRIDVYALGLVIWELMTRAHGPGDTPPDENAPPRLPPYMAPFEAEVGPIPTM      539
EmTR3      YAHSFDAYMQADIYSLGLVFWEMTRRVYVANLYGP--------EEYQLPYQDNVGPDPLI      616
EmTR1      PLLSFDVYLSTDVYALGLVLWEIWRRCTGKQ--------------YELPYYDSVPSDPNF      441
EmTR4      LGFSFEELKAADVYAFGLVLWEIFRRCLTER-GEV--------HPPHVPYWDKVSADPSF      553
                :     *:*::*:*:**:                         *          :

EmTRL      STAREMVCVKGQ----------------------------------------------RP      624
EmTR2      DKLQHYVAKLKN----------------------------------------------RP      553
EmTR3      EEMKSVVCELGI----------------------------------------------RP      630
EmTR1      LQMYRVVVLGEPYDSPCNTLVDLPLPMQVCHLCNHILVGRIGATLEAHRHRRHGGSGRRP      501
EmTR4      AEMRRVVHVEGE----------------------------------------------RP      567
                 *                                                   **

EmTRL      RLPILSEEINSLNPHQLTKATEWMRV--SDFFKNLLPECWNRDPETRLSALRIKKDLTKL      682
EmTR2      RAC-----------------SWWENDQAFVQLWETVQDSWDHDAEARITAGCIAGRIQNL      596
EmTR3      HLPLV-----------------WQTDASLRLLYHIMTECWFANPSHRLSAMRIKKDLANQ      673
EmTR1      SLTME--------------RRGSSDEEWLVRWADVIAECWHPRYTHRLSALRVRKTLTVI      547
EmTR4      PISER-----------------WQSSKVLIRCASLMQECWHAKPDVRLPILRIKKTLDET      610
                                              : :.*      *:    :   :

EmTRL      QMALSTEAKGSE------ILLFQ-------------------------------------      699
EmTR2      SQRYPPSP-----DDPASLMPFLAPT----SSINTSASSS-LEVIEVTPVA------SEP      640
EmTR3      RQQLGSPRQPGETPLPPHILRQQ-P-----SSLDALPPGV-FV-------YRSVEAASAA      719
EmTR1      EATVS-------------------------------------------------------      552
EmTR4      FSEAV--KEIGESVVPDQVIVISDPRTQADSGINSRSHGSTFQCTSVTPYYQTVVNGSIR      668


EmTRL      ------------------------------------------------------------      699
EmTR2      NPDAVPLL-----APTSTSYPDPS---AAATVGPATPAEV--------------------      672
EmTR3      SNAN-------------------TSTLETNQVGE-AEAEAEEALLASGGPTIQPPNVVAT      759
EmTR1      ------------------------------------------------------------      552
EmTR4      SPREVLALGRIMRCPSKILFSKESSTTKKLKEGEKYVAETASNLLRQEGSQLL-------      721


EmTRL      -------------     699
EmTR2      -------------     672
EmTR3      AAVSGGRGNLGYC     772
EmTR1      -------------     552
EmTR4      -------------     721
```

Supplementary Figure 1. Amino acid sequence alignment of *E. multilocularis* TGFβ receptors. Shown is a CLUSTALW alignment of EmTR2, EmTR3, EmTR4 (all characterized in this study), EmTR1 (Zavala-Gongora et al., 2006), and the predicted sequence of EmTRL

(according to Tsai et al., 2013). The colour code of protein domains as determined according to SMART is as follows: red = signal peptide, magenta = transmembrane regions, light blue = GS domains, green = kinase domain. Cysteine residues and the Cys-box in the extracellular region are marked in yellow. Red letters above the alignment indicate invariant residues important for kinase activity according to Hammaren et al. (2016). Sites of perfect alignment (*) as well as groups of strong (:) or weak (.) similarity are marked below the alignment. Numbering to the right indicates distance from start methionine. UniProt accession numbers for aligned sequences are AJ841786 (EmTR1), B7UF96 (EmTR2), CDF56918 (EmTR3), ON911572 (EmTR4), and A0A068YGF3 (EmTRL).
