# Supplementary material for: Transforming growth factor-β signalling regulates protoscolex formation in the Echinococcus multilocularis metacestode
Source: Front Cell Infect Microbiol. 2023 Mar 22;13:1153117. doi: 10.3389/fcimb.2023.1153117 (PMC10073696; doi:10.3389/fcimb.2023.1153117)
Supplement: Supplementary file 2 [file DataSheet_2.pdf]

Figure S2

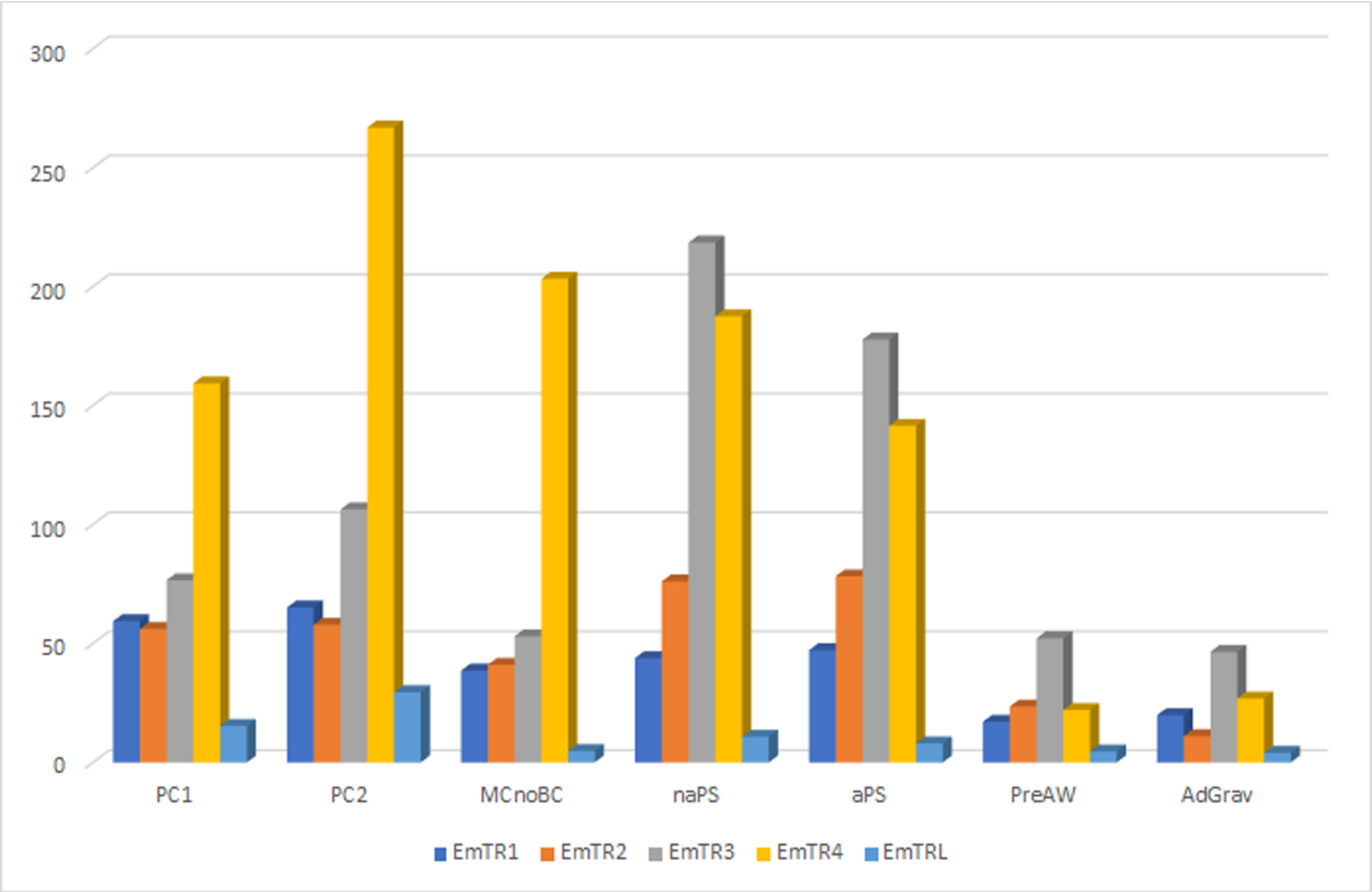

Supplementary Figure 2. Transcriptomic data on *E. multilocularis* TGFβ/BMP receptor expression in different developmental stages. Displayed are TPM (transcripts per kilobase million) values for all members of the *E. multilocularis* TGFβ/BMP receptor family characterized in this study (according to the color code below). Transcriptomic data have been collected during characerization of the *E. multilocularis* genome as reported by Tsai et al. (2013). Developmental stages are as follows: PC1, primary cells after 2 days of *in vitro* cultivation; PC2, primary cells after 7 days of *in vitro* cultivation; MCnoBC, metacestode vesicles without brood capsules; naPS, dormant (non-activated) protoscolex; aPS, pepsin/low pH activated protoscolex; PreAW, immature adult worm; AdGrav, adult worm with gravid proglottide. Please note that all transcriptomic data displayed base on only one sample (i.e. are no triplicates).
