# Supplementary material for: Transforming growth factor-β signalling regulates protoscolex formation in the Echinococcus multilocularis metacestode
Source: Front Cell Infect Microbiol. 2023 Mar 22;13:1153117. doi: 10.3389/fcimb.2023.1153117 (PMC10073696; doi:10.3389/fcimb.2023.1153117)
Supplement: Supplementary file 3 [file DataSheet_3.pdf]

Figure S3

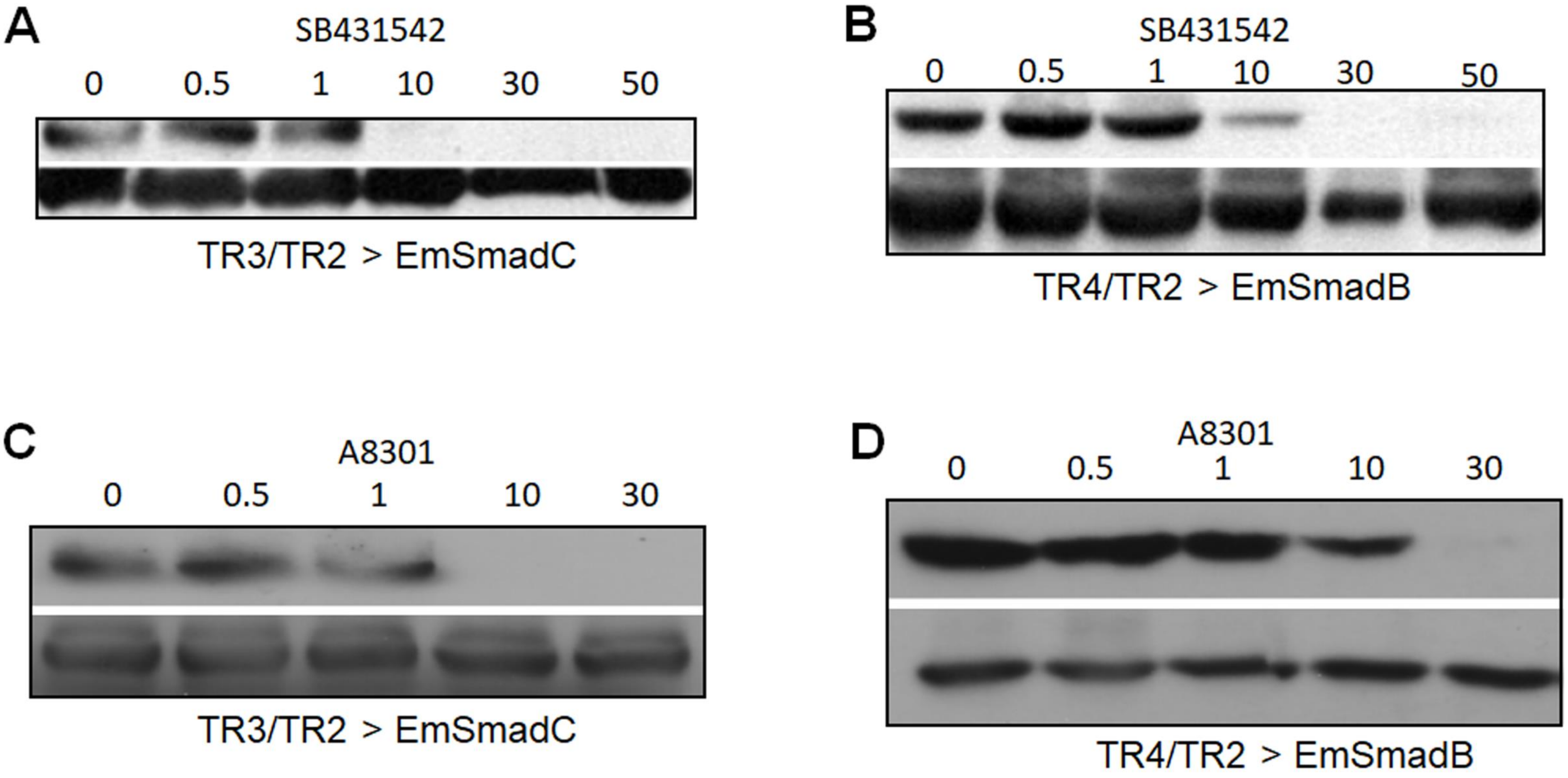

E

```
EmTR1     RETQIYTTALLSHPNILAYYASDMISRGGCTQLWLVTAYHAAGSLHDFLSTAKGVTPQCG   256
EmTR4     RETHIYSSVLLRHENILSYYASDITSRYGCTQLWIISHYHPYGSLYDFLQAH-VVDTIAA   378
TbRI      REAEIYQTVMLRHENILGFIAADNKDNGTWTQLWLVSDYHEHGSLFDYLNRY-TVTVEGM   302
EmTR3     REKEIYETATLRHSNILGFIAADNKDNGISTELWLIAEYHRLGSLYEFLQSH-AFTLPAL   477
          ** .** :. * * ***.: *:*  ..   *:**::: **  ***.::*.    .
```

Supplementary Figure 3. Inhibition of of *Echinococcus* TGFβ/BMP receptors. *Echinococcus* TGFβ/BMP receptor type I/type II combinations EmTR3/EmTR2 (A, C) and EmTR4/EmTR2 (B, D) were co-expressed with EmSmadC (A, C) and EmSmadB (B, D) in the presence of TGFβ receptor inhibitors SB431542 (A, B) or A8301 (C, D) in HEK293 T cells. In the case of EmTR4/EmTR2, human BMP2 was added at 1 nM concentration. Cell lysates were subjected to PAGE and Western blot analysis using an antibody against the phosphorylated forms of EmSmadC or EmSmadB (upper panels). As a loading control, *Echinococcus* Smads were detected using an anti-My-antibody (lower panels). Inhibitor concentrations are indicated above the Western blots (in µM). Results show representative examples of experiments performed in triplicates. (E) Amino acid sequence comparison of TGFb type I receptors around the gatekeeper residue. Displayed is a comparison between EmTR1, EmTR4, EmTR3, and the human TGFβ type I receptor (TbRI). The gatekepper residue is marked in red. Sites of perfect alignment (*) as well as groups of strong (:) or weak (.) similarity are marked below the alignment. Numbering to the right indicates distance from start methionine.
