# Supplementary material for: Transforming growth factor-β signalling regulates protoscolex formation in the Echinococcus multilocularis metacestode
Source: Front Cell Infect Microbiol. 2023 Mar 22;13:1153117. doi: 10.3389/fcimb.2023.1153117 (PMC10073696; doi:10.3389/fcimb.2023.1153117)
Supplement: Supplementary file 4 [file DataSheet_4.pdf]

Figure S4

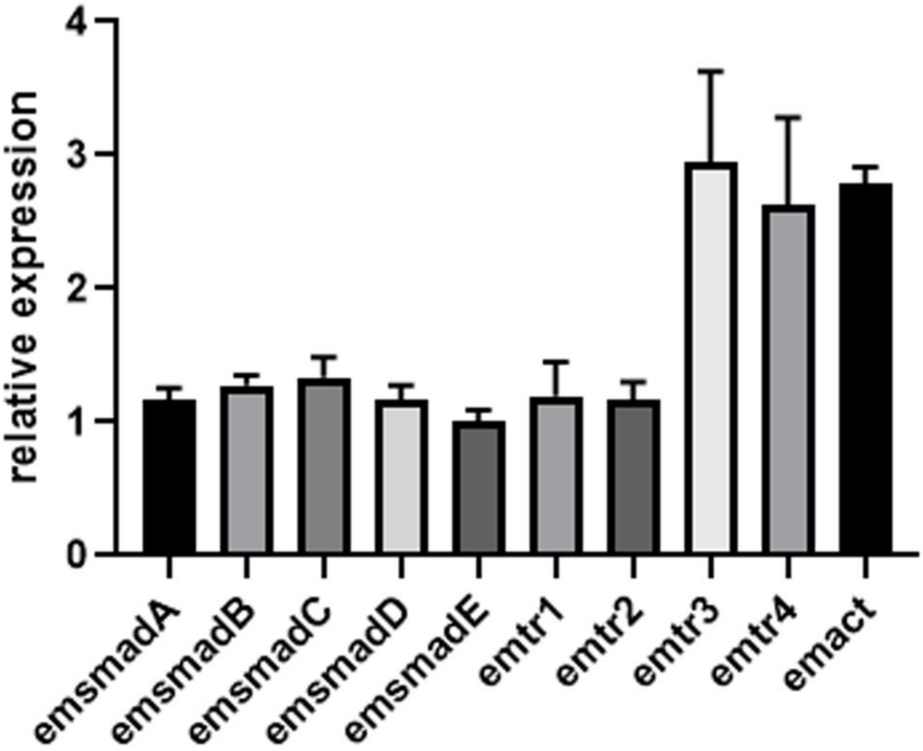

Supplementary Figure 4. RT-qPCR analysis of *Echinococcus* Smad- and TGFβ/BMP receptor encoding genes. Total RNA was isolated from axenically cultured metacestode vesicles and from vesicles of co-culture, reverse transcribed, and subjected to qPCR for Smad- and TGFβ/BMP receptor encoding genes as indicated. The constitutively expressed gene *elp* (EmuJ_000485800) was used as reference gene. Displayed is the ratio (relative expression) of each gene in co-culture versus axenically cultivated vesicles. Shown is mean and standard deviation for three independently performed analyses.
