# Supplementary material for: Transforming growth factor-β signalling regulates protoscolex formation in the Echinococcus multilocularis metacestode
Source: Front Cell Infect Microbiol. 2023 Mar 22;13:1153117. doi: 10.3389/fcimb.2023.1153117 (PMC10073696; doi:10.3389/fcimb.2023.1153117)
Supplement: Supplementary file 5 [file DataSheet_5.pdf]

Figure S5

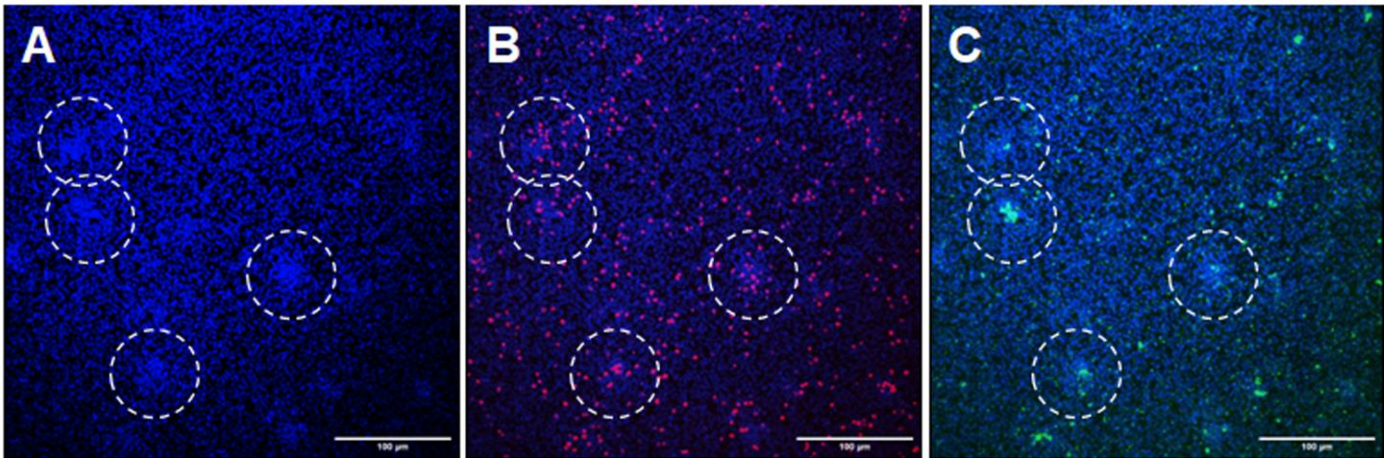

Supplementary Figure 5. Example of selection process for early BC. Displayed is a region of the metacestode GL stained with DAPI (nuclei, blue), EdU (S-phase stem cells, red), and WISH against *emact* (green). (A) shows blue channel only, (B) shows blue and red, (C) shows blue and green. Dashed circles indicate regions of interest with accumulations of total cells (blue) and proiferating stem cells (red). Bar represents 100 µm.
